# Definition and understanding of “efficiency” in healthcare provision research: a scoping review

**DOI:** 10.3389/fpubh.2024.1439788

**Published:** 2024-11-04

**Authors:** Mélanie Lötscher-Stamm, Golda Lenzin

**Affiliations:** ^1^Winterthur Institute of Health Economics, School of Management and Law, ZHAW Zurich University of Applied Sciences, Winterthur, Switzerland; ^2^Faculty of Health Sciences and Medicine, University of Lucerne, Lucerne, Switzerland

**Keywords:** efficiency, definition, measurement, healthcare provision, quality-inclusive efficiency, scoping review

## Abstract

**Background:**

With rising healthcare costs over the last decades, the concept of efficiency has gained popularity in healthcare provision research. As efficiency can be understood and measured in many different ways, it is often unclear what is meant by “efficient health systems” or “efficient healthcare providers”.

**Objectives:**

This study aims to analyze and categorize the different definitions and understandings of “efficiency” used in healthcare provision research over time.

**Methods:**

We searched five databases (Medline, Embase, CINAHL, Business Source Premier, and EconLit) to conduct a scoping review. Sources were screened independently by two researchers, using the online software Rayyan. Results are reported using PRISMA-ScR.

**Results:**

Of 1,441 individual sources identified, 389 were included in the review. Most papers (77.3%) using the term “efficiency” do not include explicit definitions or explanations of their understanding of it. Almost all papers (99.0%) are interested in productive efficiency (vs. allocative efficiency) and more specifically technical efficiency, therefore comparing the number of inputs used and outputs produced. While many papers (70.4%) include some elements of quality of care or health outcomes in their discussion, few (30.3%) include aspects of quality in their measurement of efficiency. Over the last decades, Data Envelopment Analysis has become the main method to measure efficiency. We propose a broad categorization of efficiency definitions that could be used by researchers to improve the comprehensibility and comparability of their research. Key features are the general type of efficiency, inclusion of quality or outcome information, and inclusion of cost information.

**Discussion:**

To allow for better comparability and comprehensibility, researchers in healthcare provision should state explicitly which type of efficiency they are studying. To do this, we propose to use combinations of the terms *productive efficiency*, *allocative efficiency*, *quality-inclusive efficiency*, and *cost efficiency*.

## 1 Introduction

Healthcare spending has increased in many countries over the past few decades, particularly in high-income countries (1). Policymakers and researchers are interested in understanding the underlying tendencies and finding more efficient ways to provide health care. The concept of efficiency has therefore received growing attention in healthcare provision research, leading to a variety of definitions and interpretations.

The concept of “efficiency” is rooted in economic theory. In economics, efficiency can be understood either as a productive or an allocative concept. *Productive efficiency* is interested in producing the *most* output with the least inputs (e.g., producing as many cars as possible with given materials) (2, 3). *Allocative efficiency* is interested in producing the *optimal mix* of outputs with given inputs, so that the outputs benefit society (or the company) the most, e.g., spending the available tax money on the right infrastructure renovations (3–5).

Productive efficiency can be further categorized into different subtypes. “Technical efficiency” examines the number of inputs used and outputs produced (6). Another subtype, “cost efficiency,” assigns a monetary value to the inputs used to produce the highest number of outputs at the lowest costs (7). A third subtype, “scale efficiency,” focuses on whether the analyzed economic unit (e.g., company) is the right size to produce with ideal scale effects (8, 9). Some papers applying Data Envelopment Analysis (DEA) also distinguish “allocative efficiency” as a fourth subtype of productive efficiency. In this understanding, “allocative efficiency” means using the right inputs to produce outputs with the highest possible monetary value (10), which means monetary value has to be assigned to all inputs and all outputs. In this review, these cases are assigned to the general category of productive efficiency, not allocative efficiency. Both general understandings of efficiency as well as all subtypes can also be applied to healthcare provision.

In healthcare provision research, efficiency can be analyzed from two main perspectives. Studies often either focus on health systems or healthcare providers. *Health systems* can be analyzed on an international, national, or subnational (e.g., state or regional) level. The efficiency goal of health system studies is often to provide a higher level of care with the same set of resources. Another perspective can be to provide the same level of care to a larger or older population with the same resources as before (11–13). Studies interested in *healthcare providers* often focus on one type of provider, such as hospitals, primary care centers, or nursing homes. The efficiency goal of healthcare provider studies is often to reduce costs by providing the same level of care with fewer resources (14–16).

Several systematic literature reviews and scoping reviews have been conducted on specific aspects of efficiency. These include reviews on efficiency in nursing homes (17), efficiency in primary care in high-income countries (18), and efficiency of health systems (19). Others have focused on the different methodologies of efficiency measurement, for example on the application of different frontier techniques (20) or specifically on the inputs and outputs used in efficiency models (21). Another review was conducted on different measures implemented to improve efficiency in health care (22).

The existing reviews have highlighted the diverse interpretations and understandings of the term “efficiency” being used in healthcare provision research. This diversity can sometimes be problematic: If research papers have different underlying understandings of efficiency, comparability between research on the topic might be very limited. Especially in healthcare provision research, where comparison between countries, regions, or providers is frequently performed, this ambiguity can be detrimental to the quality of research and the resulting policy recommendations. So far, there has been no review incorporating different settings and levels of interest, focusing on the concept of efficiency itself rather than on the most efficient ways to organize a specific setting (e.g., nursing homes) or on specific methodologies.

This paper therefore aims to answer the following questions:

1. How has the term “efficiency” been defined and understood in the scientific literature on health care provision?
2. Have the definitions and understandings changed over time?
3. How can the definitions and understandings be broadly categorized for easier comparability?

By “definitions and understandings,” we refer to both explicit definitions provided in sources and implicit definitions, where the underlying definition needed to be derived from the way efficiency was measured.

The study was designed using a scoping review approach. This type of review was chosen because the goal of the paper is to clarify a concept, which is one of the indications for a scoping review rather than a systematic literature review (23, 24). However, while the topic of this review lends itself to a scoping review, the methods used to identify, screen, and include or exclude articles for analysis are equivalent to a systematic literature review.

## 2 Materials and methods

This scoping review was conducted in accordance with the framework for scoping reviews by Arksey and O’Malley (25) and is reported under the Preferred Reporting Items for Systematic Reviews and Metaanalyses extension for Scoping Reviews (PRISMA-ScR; checklist available in the Supplementary Table 1). In some aspects, the guidelines of the Joanna Briggs Institute (JBI) methodology for scoping reviews (26) were followed for more detailed orientation. The research protocol for this review was not registered.

### 2.1 Identifying relevant studies

The authors conducted a systematic literature search in five literature databases (Medline, Embase, CINAHL, Business Source Premier, and EconLit). Results in English, French, and German were included. Only original research articles published in scientific journals were considered. No limits were set for the publication date of sources.

Preliminary search terms were developed by the research team. For the final design of the search strategy and the realization of the search, a scientific librarian of the medical library of the University of Zurich was consulted. The main keywords used included efficiency, inefficiency, and different keywords related to providers (hospital, primary care, health center, outpatient, etc.). To refine the search to studies that provide definitions or measurements of efficiency, supplementary terms such as defin*, measur*, or indicat* were added. Boolean operators such as AND, OR, and NOT were applied. For full search terms, see Supplementary Table 2. The most recent search was executed on January 25th, 2022. The results of the search have been saved and managed in EndNote 20 (27).

While this study focuses on efficiency, the related term “inefficiency” was also searched for and is used in some of the included sources. Efficiency and inefficiency describe the two opposite facets of the same concept, e.g., studies searching for inefficient providers usually search for the least efficient ones. Studies might use “inefficiency” as a keyword but then provide definitions and measurements for “efficiency” [see for example (28, 29)] or give negative formulations of efficiency definitions for inefficiency [see for example (30, 31)]. This scoping review, therefore, also indirectly applies to research on inefficiency.

### 2.2 Selection of sources of evidence

The selection process involved (i) removal of duplicates, (ii) title/abstract screening, and (iii) full-text screening. Duplicates were removed automatically with the corresponding function of EndNote and an additional manual search for duplicates in the EndNote library. The screening was performed with the online software Rayyan (32).

For the title/abstract screening, sources were included or excluded based on pre-defined criteria. The screening guide is provided in Supplementary Table 3. The main criteria in this first screening stage were language, publication type, setting, and content. The “setting” criteria excluded studies on efficiency in dental care, military health care, and medical labs as these settings are or can be quite different from other healthcare provision settings. The “content” criteria excluded studies interested in other types of efficiency than economic efficiency, such as energy efficiency, treatment efficiency, imaging efficiency, etc. It also excluded medical studies focusing on a specific condition, screening, intervention, or medication, as this type of efficiency is not the focus of this review. A 93.4% agreement between the two screeners was reached in this first step of title/abstract screening. Disagreements were resolved by discussion.

For the full-text screening, a second screening guide was developed pre-screening (also provided in Supplementary Table 3). The criteria of the first stage were supplemented by criteria on “definition.” Sources without an explicit or implicit definition of their understanding of efficiency were excluded. This mainly concerned studies using the term in the abstract but not in the body of the paper. Availability of the full text to the authors was another additional criterion. A 94.7% agreement between the screeners was reached after the first round of full-text screening. Conflicts were resolved by a separate, blinded re-screening of all contested articles by both MLS and GL. If the disagreement persisted, consensus was reached by discussion.

**Figure 1:**
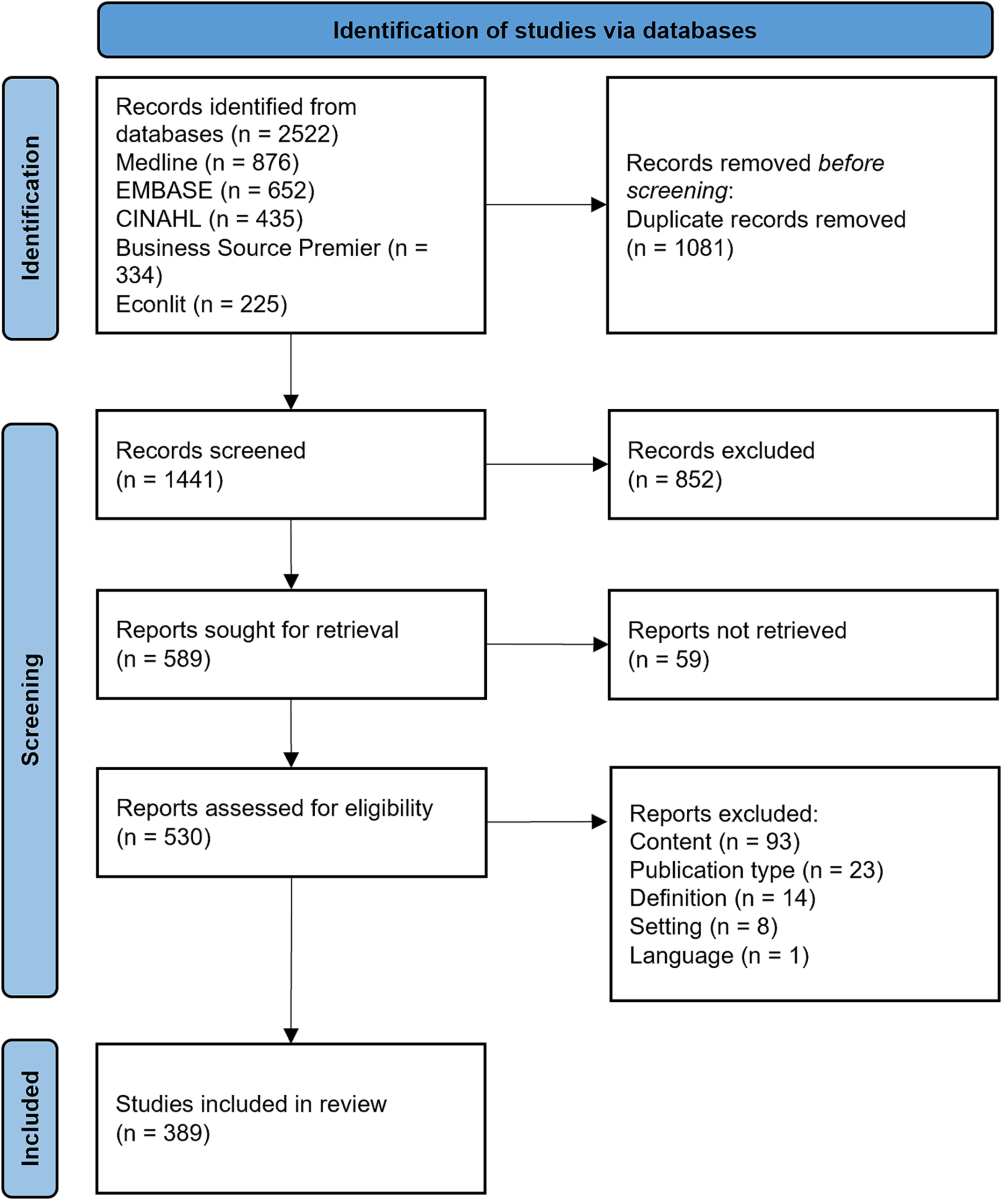
Results of the systematic search and screening process, using the Preferred Reporting Items for Systematic Reviews and Meta-Analyses (PRISMA) flow diagram.

The results of the search and the study inclusion process are presented in Figure 1 in a PRISMA flow diagram (33). Reasons for the exclusion of sources at full-text screening are reported in the diagram.

### 2.3 Data extraction and analysis

Each author extracted information from half of the included sources into a charting template developed in Microsoft Excel (Excel 365, 2022). After charting, MLS conducted some quality control measures by searching the charting document for cells that had mistakenly not been filled. The missing information was then extracted directly from the corresponding source. Additionally, we ensured that, depending on the type of study, the correct cells were filled and/or left empty (e.g., when the method “DEA+” was selected, the cells on “inputs” and “outputs” should be filled, but the cell on “indicators” should be empty).

Extracted information included author, year, country of interest, level of interest (health system or health provider), study setting, direct or indirect definition of efficiency, type and subtype of efficiency studied, method used to quantify efficiency, input variables, output variables, indicators used, inclusion of quality of care or health outcomes in the discussion (yes/no), inclusion of quality of care or health outcomes in the efficiency definition (yes/no). Some categories were simplified for the analysis. One example concerns the countries of interest, which were categorized into the five UN world regions (34). The extracted data was handled and analyzed in Microsoft Excel and Stata (release 17) (35).

## 3 Results

### 3.1 Selection of sources of evidence

The initial search resulted in 2522 studies retrieved from the different databases, which were reduced to 1,441 studies after the removal of duplicates. After the screening of titles and abstracts, 589 studies were sought for full-text retrieval. 530 studies could be retrieved and were included in the full-text screening. Finally, 389 studies were included in the review. Reasons for exclusions at this stage are shown in Figure 1. All included sources are detailed in Supplementary Table 4.

Some of the included studies use multiple definitions or measurements of efficiency. This means that some analyses (e.g., general study characteristics) were conducted on the level of studies (*n* = 389). Other analyses (type of efficiency, method used, inputs and outputs used, etc.) are performed on the level of definition because two different versions can be used in one single study. Therefore, these analyses are performed on the slightly larger sample of definitions (*n* = 405). The Excel documents with the extracted characteristics are available in Supplementary Data Sheets 1, 2.

### 3.2 Results on the level of studies (*n* = 389)

#### 3.2.1 General study characteristics

The included studies were published between 1974 and 2022. Table 1 summarizes the general study characteristics. 90.5% (*n* = 352) of the studies focus on a single country, while 9.5% (*n* = 37) are interested in multiple countries (globally or within a specific region). 32.7% (*n* = 115) of the studies interested in a single country focus on countries in Asia, 28.1% (*n* = 99) each on countries in the Americas and Europe, 8.5% (*n* = 30) on countries in Africa, and 2.6% (*n* = 9) on countries in Oceania.

**Table 1:** General characteristics of included studies.

Variable		Frequency (%)
Year of publication	1970–1979	3 (0.8%)
	1980–1989	8 (2.1%)
	1990–1999	34 (8.7%)
	2000–2009	74 (19.0%)
	2010–2019	198 (50.9%)
	2020–2022	72 (18.5%)
Geographical focus	Single country	352 (90.5%)
	Multiple countries	37 (9.5%)
U.N. world regions (single country studies)	Africa	30 (8.5%)
	Americas	99 (28.1%)
	Asia	115 (32.7%)
	Europe	99 (28.1%)
	Oceania	9 (2.6%)
Level of interest	Health providers	321 (82.0%)
	Health systems	69 (18.0%)
Provider settings	Hospitals	255 (79.4% of provider studies)
	Primary care providers	53 (16.5%)
	Nursing homes	6 (1.9%)
	Secondary care (excl. hospitals)	4 (1.3%)
	Other	3 (0.9%)
Health system settings	International analyses	32 (46.4% of health system studies)
	National analyses	15 (21.7%)
	Sub-national analyses	22 (31.9%)
Quality of care and health outcomes	Quality included in the discussion	274 (70.4%)
	Quality included in the definition	118 (30.3%)

#### 3.2.2 Levels and settings of interest

Most included studies analyze health providers (82%, *n* = 321), and only a minority analyze health systems (18%, *n* = 69). One study covers both provider and health systems and is included in the calculations for both settings. Regional variations exist: research concerning Africa, the Americas, Asia, and Europe mainly covers health providers (all over 88%). Studies focused on Oceania are more balanced between the levels, with 44.4% (*n* = 4) analyzing providers and 55.6% (*n* = 5) analyzing health systems. Studies done in an international setting are predominantly interested in health systems (81.1%, *n* = 30).

Of the studies analyzing providers, nearly 80% (*n* = 255) are interested in hospitals, 16.5% (*n* = 53) in primary care, and 4.1% (*n* = 13) in nursing homes, pharmacies, and other secondary care. Of the studies focusing on health systems, 46.4% (*n* = 32) conduct international analyses, 21.7% (*n* = 15) conduct analyses on a national, and 31.9% (*n* = 22) on a sub-national level.

95.9% (*n* = 373) of all papers use only one definition or measurement of efficiency.

#### 3.2.3 Inclusion of quality of care or health outcomes

While there is no universal agreement on how to define or measure the quality of healthcare provision, we can generally divide healthcare quality into structural, process, and outcome quality (36). Most of the quality indicators used in the studies covered in this review focus on outcome quality (e.g., complication rates, unplanned readmission rates, or number of pressure ulcers). As we also cover health system analyses, more general health outcome measures (such as life expectancy or child mortality rates), which are far more comprehensive than the aforementioned examples of quality of care indicators, are also of interest.

We evaluated each source to determine whether any elements of quality of care or health outcomes were included in the discussion and quantification of efficiency. This analysis was performed to understand whether “efficiency” is understood as a purely economic concept (number or cost of inputs and outputs) or whether healthcare provision researchers deem providers and health systems to be “most efficient” if they deliver health care with the fewest inputs and best health outcomes for the treated patients or population. For “quality included in discussion,” statements as general as “quality of care is assumed to be equal in all analyzed hospitals” or “we wanted to include health outcomes, but there was no data available” were accepted. As a result, we created a broad indicator of whether quality of care or health outcomes were even slightly considered by the authors of the included sources. In contrast, we marked “quality included in definition” only when elements of quality of care or health outcomes were utilized in the calculation of efficiency.

70.4% (*n* = 274) of all included studies mention aspects of quality of care or health outcomes in their discussion. This share reaches 91.3% (*n* = 63) of studies covering health systems, while only 66.0% (*n* = 212) of provider-focused studies discuss aspects of quality of care or health outcomes.

However, only 30.3% (*n* = 118) of all studies include elements of quality of care or health outcome measures in their definitions or measurements of “efficiency.” This represents 43.1% of the studies that discussed elements of quality or outcomes. Studies related to health systems more frequently incorporate quality of care or health outcomes into their efficiency definitions or measurements (73.9%, *n* = 51 vs. 21.2%, *n* = 68 for studies focusing on providers). This is expected as these analyses often involve population health outcomes such as life expectancy at birth or child mortality.

**Figure 2:**
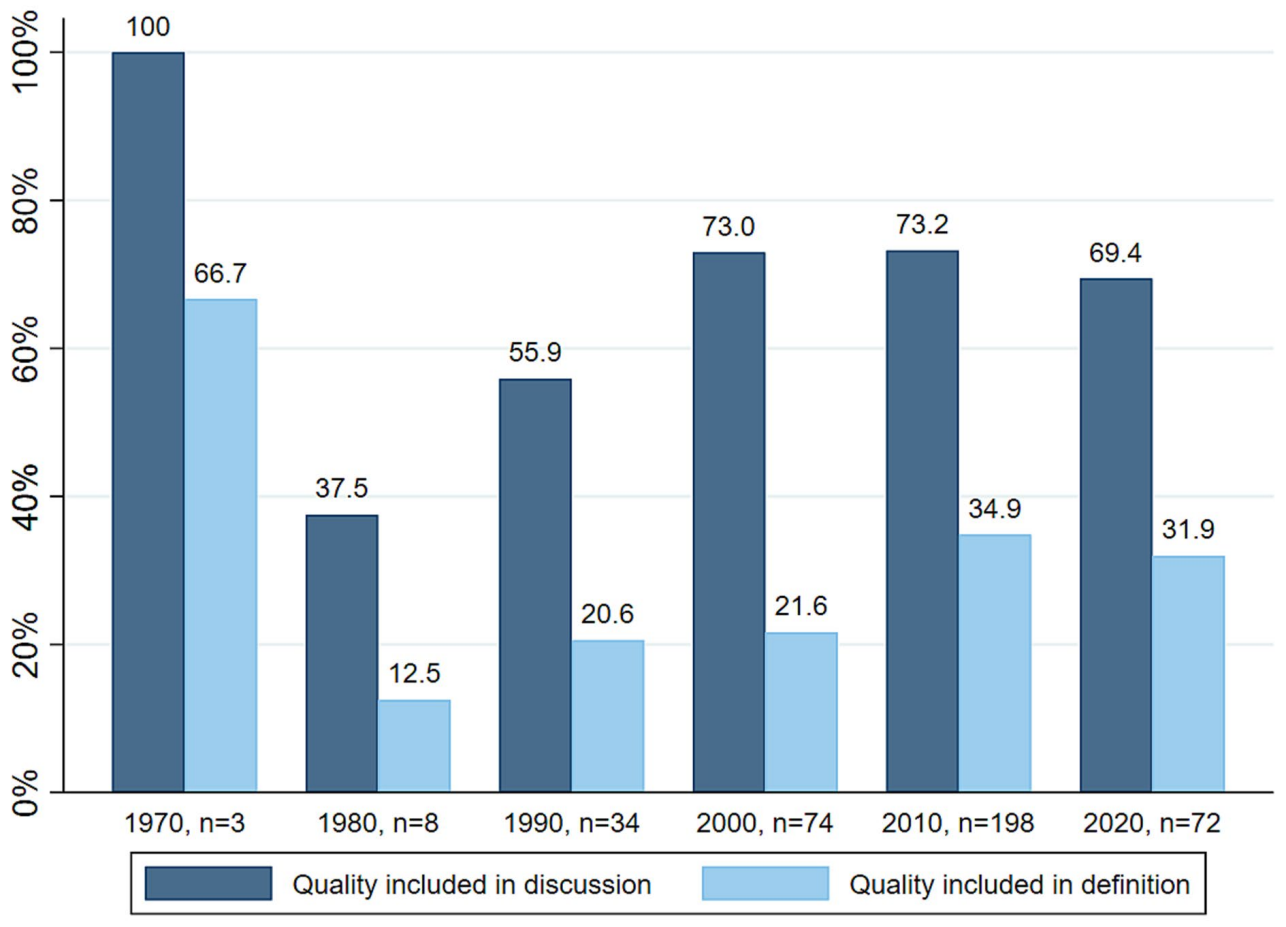
Proportion of studies including quality of care or health outcomes in their discussion and definition, per decade.

Figure 2 illustrates the differences in quality inclusion over time. Apart from the three studies published in the 1970s, all of which discussed aspects of quality of care or health outcomes, we can see an increase in the proportion of studies discussing such elements over time (37.5%, *n* = 3 in the 1980s, to 73.2%, *n* = 145, in the 2010s). Similarly, the proportion of studies including quality of care or health outcomes in their definition and measurement of efficiency increased from 12.5% (*n* = 1) in the 1980s to 34.8% (*n* = 69) in the last full decade analyzed (2010–2019).

### 3.3 Results on the level of definitions (*n* = 405)

#### 3.3.1 Direct or indirect definitions

Table 2 summarizes the definitions and methods used in the included studies. Only in 22.7% (*n* = 92) of all cases is an explicit definition of “efficiency” included. The authors of the included studies typically do not provide a clear definition of the term, requiring the reader to analyze how efficiency is measured in the study to understand the intended meaning.

**Table 2:** Definitions and methods used in included studies.

Variable		Frequency (%)
Explicit definition of efficiency	Included	92 (22.7%)
	Not included	313 (77.3%)
Efficiency type	Productive efficiency	401 (99.0%)
	Allocative efficiency	2 (0.5%)
	Both	2 (0.5%)
Subtypes of productive efficiency	Technical efficiency	319 (93.0% of all studies with just one subtype)
	Cost efficiency	21 (6.1%)
	Scale efficiency	2 (0.6%)
	Social efficiency	1 (0.3%)
Method used	DEA (+)	300 (74.1%)
	SFA (+)	41 (10.1%)
	Indicators	41 (10.1%)
	Other (OLS models, Pabón Lasso models, etc.)	21 (5.2%)
	None	2 (0.5%)

#### 3.3.2 Type and subtype of efficiency

99.0% (*n* = 401) of the definitions are based on a productive understanding of efficiency. Only a small percentage (0.5%, *n* = 2 each) are based on an allocative understanding of efficiency or both types.

Most definitions and measurements of efficiency (84.7%, *n* = 343) use only one subtype of efficiency, while 13.6% (*n* = 55) of them include multiple subtypes such as technical, scale, and cost efficiency. Some definitions (1.7%, *n* = 7) do not include a subtype.

Among the definitions and measurements including only one subtype of efficiency, the vast majority (93.0%, *n* = 319) relies on technical efficiency, while only a few rely on cost efficiency (6.1%, *n* = 21) and even fewer rely on scale efficiency (0.6%, *n* = 2) or what the authors called “social efficiency” (0.3%, *n* = 1).

#### 3.3.3 Methods used to quantify or measure efficiency

In most cases (74.1%, *n* = 300), efficiency is quantified by DEA or a DEA-based method [DEA (+)]. DEA is a non-parametric linear programming method used to determine and compare the efficiency of so-called “decision-making units,” which in healthcare can be different types of providers or health systems. Units are compared based on the ratio of a weighted sum of the outputs of the unit to a weighted sum of its inputs (37). In some cases (10.1%, *n* = 41), efficiency is quantified by Stochastic Frontier Analysis (SFA) or SFA-based methods [SFA (+)]. SFA is a parametric method, calculating a best-practice frontier based on multiple inputs and a singular output using least squares methods (38, 39). Indicators are used quite frequently (10.1%, *n* = 41), while alternative methods such as OLS models, mixed effect models, Pabón Lasso models, or order-m-estimators are used less frequently (5.2%, *n* = 21). In two instances, a study proposed an explicit definition of efficiency without any quantification or measurement (0.5%, *n* = 2).

**Figure 3:**
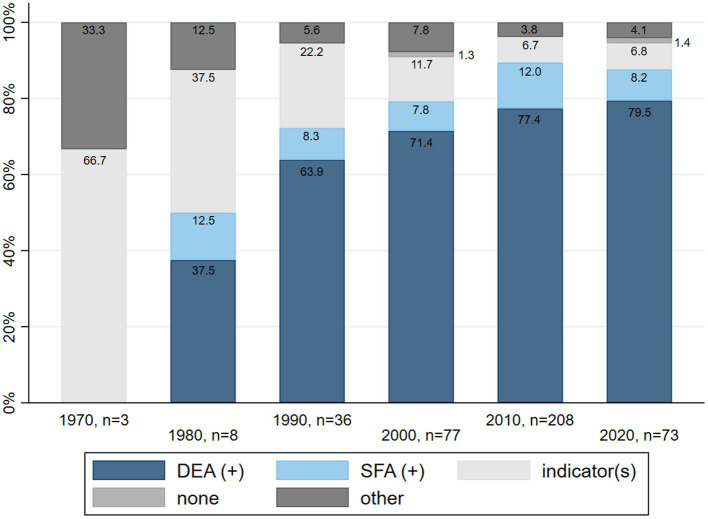
Relative frequency of methods used to quantify efficiency in the included studies, per decade.

Figure 3 shows that DEA has become the main method used to quantify efficiency in healthcare provision research.

#### 3.3.4 DEA (+)

DEA models can generally be categorized as *input-oriented models*, trying to minimize inputs for constant outputs, or *output-oriented models*, aiming to maximize outputs for constant inputs. Among studies that employ DEA models, the majority (58.7%, *n* = 176) use input-oriented models, while 25.3% (*n* = 76) use output-oriented models, and 6% (*n* = 18) use either both or non-oriented models. In 10% (*n* = 30) of all DEA applications, information about the orientation of the model could not be extracted.

DEA applications at the provider level mostly use input-oriented models (62.8%, *n* = 153 as opposed to 21.3%, *n* = 52 output-oriented models), whereas DEA applications at the health system level use both input-oriented and output-oriented models with equal intensity (41.8%, *n* = 23 each).

To analyze the inputs and outputs used in DEA papers, they were grouped into broad categories. Table 3 provides details on the inputs and outputs used. The input categories included labor, costs, supplies, services provided, capital, case complexity, and, exclusively for health systems, the number of medical institutions. Labor-related inputs (86.3% of all DEA papers, *n* = 259), supply-related inputs (69.9%, *n* = 210) such as hospital beds, and cost-related inputs (36.5%, *n* = 109) were the most frequently used categories.

**Table 3:** Categories of inputs and outputs used in DEA (+) studies.

	Provider (*n* = 244)	Health systems (*n* = 55)	Total (*n* = 299)
Inputs			
Labor	222 (91.0%)	36 (65.5%)	258 (86.3%)
Costs	80 (32.8%)	29 (52.7%)	109 (36.5%)
Supplies	178 (73.0%)	31 (56.4%)	209 (69.9%)
Services provided	15 (6.2%)	1 (1.8%)	16 (5.4%)
Capital	26 (10.7%)	4 (7.3%)	30 (10.0%)
Case complexity	12 (4.9%)	0 (0%)	12 (4.0%)
No of institutions	6 (2.5%)	5 (9.1%)	11 (3.7%)
Outputs			
Services provided	232 (95.1%)	25 (45.6%)	257 (86.0%)
Quality/outcomes	39 (16.0%)	36 (65.5%)	75 (25.1%)
Case complexity	34 (14.0%)	4 (7.3%)	38 (12.7%)
Revenue	20 (8.2%)	1 (1.8%)	21 (7.0%)
Training	11 (4.5%)	1 (1.8%)	12 (4.0%)

The outputs used in DEA papers were categorized into services provided, quality/outcomes, case complexity, revenue, and training. Services provided (86.0% of all DEA papers, *n* = 258) and outcomes (25.1%, *n* = 76) were the most frequently used output categories. The category of quality/outcomes covers many different variables, such as, for example, mortality rates, life expectancy, and percentage of births discharged alive, but also unplanned readmission rates or long-term complication rates.

#### 3.3.5 Ratios and indicators

Efficiency was measured using one or multiple indicators in 41 cases. 48.8% (*n* = 20) of those studies used cost indicators, while 41.5% (*n* = 17) used length of stay (LOS) indicators. Additional or other indicators were used in *n* = 27 cases. Studies focusing on health systems used additional indicators such as avoidable mortality rate, primary care coverage rate, or improvements in life expectancy. Studies focusing on providers utilized additional indicators such as staff per occupied bed, operations per month per operating room, or the bed occupancy rate.

### 3.4 Categorization of definitions

**Figure 4:**
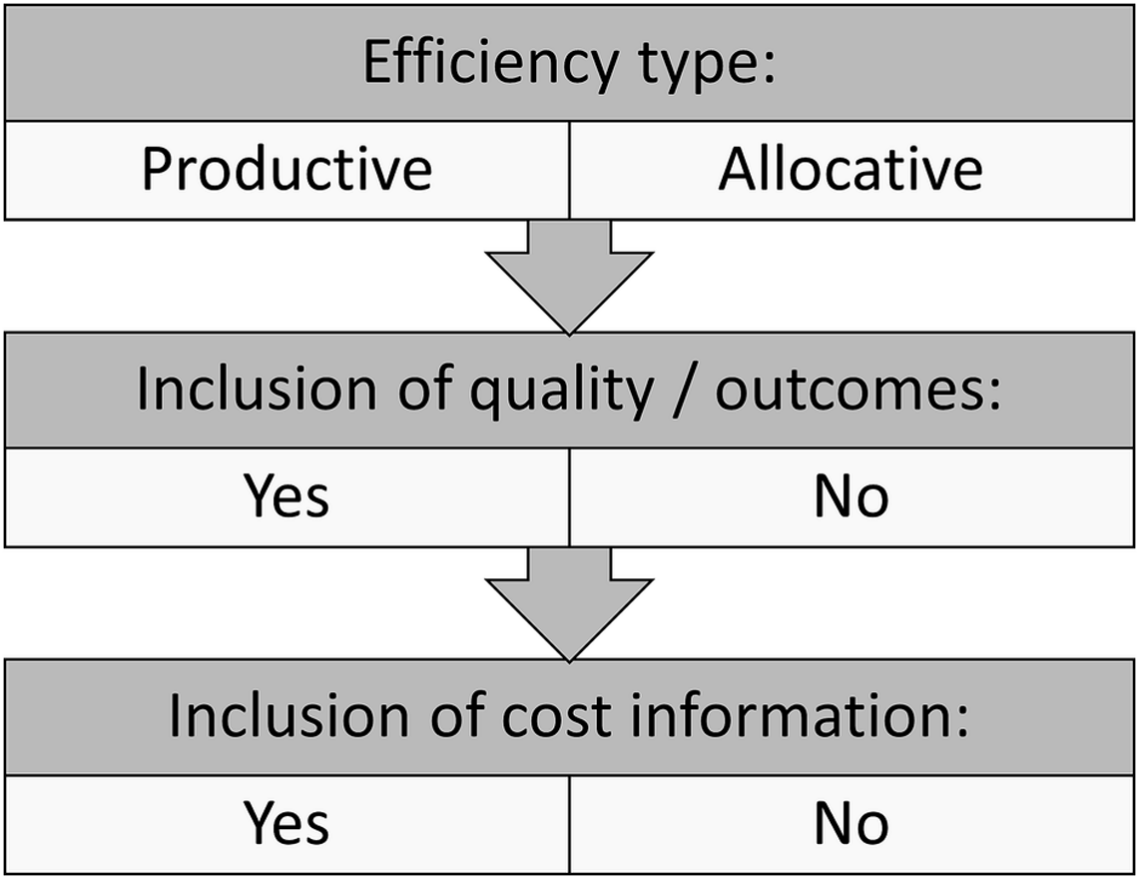
Gross categorization of efficiency definitions in healthcare provision research.

While a detailed analysis of the definition of efficiency used in healthcare provision research may not always be necessary, we believe that a broad categorization of definitions would facilitate comparability and comprehensibility of efficiency research. As such, one goal of this review was to propose a broad categorization of the definitions used in the healthcare literature over the last decades. We suggest that when designing efficiency studies or projects aimed at improving efficiency in health care, the underlying understanding of efficiency should be stated according to the key features presented in Figure 4.

The three key features of our categorization are the general type of efficiency (productive vs. allocative), the inclusion of quality of care or health outcome measures, and the inclusion of cost information. Although additional differences of methodological and other nature exist, we consider these three features to be the most distinctive. Figure 4 can be applied to different levels of analysis (health care systems vs. health care providers), different clinical settings, and different types of research. It could be used as a support tool both for designing efficiency studies and for communicating their results.

Communication is facilitated by the use of established and easily comprehensible terminology. The use of a specific term for each possible combination of the key features in Figure 4 would complicate, rather than simplify, the communication of efficiency studies and their results. Therefore, we propose to use combinations of well-known terms to effectively communicate the type of efficiency studied:

- *productive efficiency* and *allocative efficiency* for the general type of efficiency
- *cost efficiency* if cost information is included

To the best of our knowledge, no such well-established term exists for efficiency which includes aspects of quality of care or health outcomes. We would therefore propose the following terminology:

- *quality-inclusive efficiency* if information on quality of care or health outcomes is included

Consequently, for studies including cost and/or quality information, two or even three terms would be applicable. To ensure the greatest clarity and comprehensibility, we would recommend using all applying terms in the respective manuscript, and potentially as keywords.

## 4 Discussion

### 4.1 Summary of results

The purpose of this study was to investigate how the term “efficiency” has been defined in the scientific literature on healthcare provision, how these definitions have changed over time, and how they can be categorized for better comparability.

Our analysis shows that most papers using the term “efficiency” in healthcare provision research fail to provide explicit definitions or explanations of the concept. As a result, readers need to infer the authors’ understanding of efficiency based on the quantifications provided.

Most papers are interested in productive rather than allocative efficiency. This is true regardless of the level of interest (health systems or specific providers). Most papers focus on technical efficiency (the relation between the number of inputs and outputs), while only a few use cost efficiency, where cost information on inputs is needed. Efficiency is consistently understood as a comparative term, indicating that it is impossible to determine efficiency without one or more comparators. Comparators can consist of other countries, regions of the same country, or other providers. Although many papers discuss some aspects of quality of care or health outcomes, only a few include quality – as defined for this review—in their measurement of efficiency. Overall, efficiency is mostly understood as a concept that compares the ratio between the number of inputs used and outputs produced, not including quality measures or health outcomes, and only rarely including costs of the inputs.

One main change over time concerns the methodology used to quantify efficiency. Over the past 30 years, research on efficiency in health care has become strongly focused on applications of Data Envelopment Analysis (DEA) or Stochastic Frontier Analysis (SFA). The use of efficiency indicators such as “length of stay” or “expenses per standard patient” on their own has decreased, even though these indicators are now commonly integrated as inputs or outputs in DEA applications. A further change over time includes the rise in the proportion of studies incorporating quality or outcome elements in their discussion and definition of efficiency. Excluding the three studies from the 1970s, which all incorporated quality of care elements, the proportion of studies doing so has continued to steadily increase from the 1980s to the last full decade analyzed (2010–2019). This could indicate that the quality aspect of health care is becoming more prominent and that efficiency is developing into a more comprehensive concept.

Our review results are consistent with other reviews conducted on efficiency in specific settings. In Neri et al.’s (18) study on primary care efficiency, all included studies focused on technical efficiency, with cost efficiency and other subtypes only rarely being investigated. Regarding the incorporation of quality and outcome considerations into efficiency discussions, Hussey et al. (22) discovered that almost none of the efficiency measures analyzed in their scoping review included quality of care. From their perspective, this means that efficiency measures only account for the cost of care but not for what they define as ‘true efficiency’, which would require the inclusion of both quality and cost (22). It should be noted that while many authors view quality of care as a component of efficiency, others consider efficiency and quality of care to be two concurrent concepts (40). A recently published review on healthcare quality in nonparametric efficiency studies goes into more detail on how exactly quality is incorporated in the calculation of efficiency scores (41). Regarding the methods used to measure the concept of efficiency, our study’s findings are consistent with previous research conducted on health system efficiency. According to Mbau et al. (19), the most common method is DEA, followed by SFA. Hafidz et al. (42) similarly reported that DEA is the most commonly used method to measure efficiency in studies on healthcare facilities in low- and middle-income countries since the year 2000.

After the analysis of the included studies, we proposed a categorization of definitions and understandings of efficiency along three key features: general type of efficiency (productive vs. allocative), inclusion of quality of care or health outcome measures, and inclusion of cost information. We also proposed that researchers should use the following terms in combination to clearly communicate the type of efficiency analyzed in their research: *productive* vs. *allocative efficiency*, *quality-inclusive efficiency*, and *cost efficiency*.

As an example of the usefulness of our categorization, consider an efficiency study on primary care practices. Is the efficiency goal to reduce the number of inputs (e.g., GPs) with constant outputs (e.g., patients treated per week)? Or is the efficiency goal to improve patient outcomes (e.g., patient satisfaction) with the same cost of inputs (e.g., labor costs)? These two examples have vastly different understandings of efficiency and therefore different goals and implications. Findings from efficiency studies using technical efficiency without the inclusion of elements of quality of care or health outcomes (which, as we have shown, represent the majority of efficiency studies) might be less directly applicable to healthcare providers or political stakeholders who would include aspects of quality in their understanding of efficiency. An explicit statement of the general understanding of efficiency used in studies or projects according to the proposed key features and terms would facilitate the assessment of the comparability of studies as well as the practical applicability of study results. In addition, it would make the communication of results to non-scientific stakeholders and policymakers more precise and more comprehensible.

### 4.2 Limitations

Two limitations of this study should be noted. The first limitation results from the time that passed between the final database searches in January 2022 and the final submission of the paper in Spring 2024. Because of this delay, papers published after January 25th, 2022 were not considered in the review, resulting in the exclusion of the most recent papers on healthcare efficiency. As this scoping review is interested in long-term developments and covers five decades (1970 to 2020), we do not believe that re-running the search for two more years would add any substantial benefits compared to the additional effort. The authors do not expect this limitation to strongly influence the results of the paper. Nonetheless, efficiency studies published over the last two years are missing from this review [see for example (43–45)].

The second limitation stems from the lack of additional ways used to identify relevant sources. Although it was planned to search for further sources in the reference sections of included studies according to the JBI guidelines (26), it was not possible to do so due to the considerable number of included studies based on database searches alone. It is likely that our database search did not detect some sources of evidence that would fit our inclusion criteria and could have been found through manual reference searching. As the included studies from the database search alone cover five decades, many different countries, and topics, we do not believe that the lack of additional search methods strongly influences the results of this broad and conceptual scoping review. Other types of sources (reports, non-scientific articles, etc.) were not searched for as the research questions of this scoping review are specifically interested in scientific research.

## 5 Conclusion

This review has shown that many different definitions and understandings of “efficiency” have been used in the scientific literature on healthcare provision. Interestingly, many papers do not include an explicit definition of “efficiency.” In most cases, efficiency is understood as a productive term, interested in the number of inputs used and the number of outputs produced. There is considerable variation in terms of whether quality of care and health outcomes are seen as part of efficiency and if so, how they are included in the quantification of efficiency. While different methods have been used to quantify efficiency over time, Data Envelopment Analysis has become increasingly dominant.

Since efficiency is often used without an explicit definition, we propose a broad categorization of efficiency definitions that could be used by researchers to improve the comprehensibility and comparability of their research. Key features are the type of efficiency, inclusion of quality information, and inclusion of cost information. Additionally, as quality of care is rarely incorporated in efficiency quantifications, this should be stated clearly by researchers when expecting their results to influence policy decisions.

## Data Availability

The original contributions presented in the study are included in the article/Supplementary material, further inquiries can be directed to the corresponding author.

## Supplementary material

Supplementary Table 1 PRISMA-ScR Checklist.

Supplementary Table 2 Full search strategy.

Supplementary Table 3 Screening guide.

Supplementary Table 4 Full reference list.

Supplementary Data Sheet 1 Extraction - level of papers.

Supplementary Data Sheet 2 Extraction - level of definitions.
