# Supplementary material for: Definition and understanding of “efficiency” in healthcare provision research: a scoping review
Source: Front Public Health. 2024 Nov 4;12:1439788. doi: 10.3389/fpubh.2024.1439788 (PMC11571063; doi:10.3389/fpubh.2024.1439788)
Supplement: Supplementary Table 2 — Full search strategy. [file Table_2.DOCX]

Supplementary Material 2: Full search strategy for one database (EMBASE)

Embase Session Results (25 Jan 2022)

| No. | Query | Results |
| --- | --- | --- |
| #6 | #1 AND #2 AND #3 AND ([english]/lim OR [french]/lim OR [german]/lim) NOT [conference abstract]/lim | 652 |
| #5 | #1 AND #2 AND #3 AND ([english]/lim OR [french]/lim OR [german]/lim) | 776 |
| #4 | #1 AND #2 AND #3 | 827 |
| #3 | ((defin* OR measur* OR scor* OR index OR indicat*) NEAR/3 efficiency):ti,ab | 17412 |
| #2 | 'health care'/exp OR 'health care organization'/exp OR 'hospital management'/exp OR (((health OR primary OR outpatient OR ambulatory) NEAR/3 (care OR service* OR therapy OR treatment)):ti,ab) OR hospital*:ti,ab OR (((general OR family) NEAR/3 practic*):ti,ab) | 8386030 |
| #1 | 'organizational efficiency'/exp OR 'economic efficiency'/exp OR 'cost effectiveness analysis'/exp OR (('health care cost'/exp/mj OR cost:ti OR economic:ti OR organizational:ti OR practice:ti OR health*:ti OR hospital*:ti) AND (efficiency:ti OR inefficiency:ti)) OR (((cost OR economic OR organizational OR practice OR health OR  hospital*) NEAR/3 (efficiency OR inefficiency)):ab,kw) | 181268 |
