# Supplementary material for: Definition and understanding of “efficiency” in healthcare provision research: a scoping review
Source: Front Public Health. 2024 Nov 4;12:1439788. doi: 10.3389/fpubh.2024.1439788 (PMC11571063; doi:10.3389/fpubh.2024.1439788)
Supplement: Supplementary Table 3 — Screening guide. [file Table_3.DOCX]

Supplementary Material 3: Screening guide

# Screening guide for the title/abstract screening:

| **Criterion** | **Include** | **Exclude** |
| --- | --- | --- |
| Language | - Title and/or abstract are in English, German or French | - Title is not English, German or French  - Abstract is not English, German or French  - Abstract includes hints that full text is in another language (like “abstract in english”) |
| Publication type | - Entry is an original scientific article  - Entry is a book chapter | - Entry is a whole book  - Entry is a systematic review or other type of review  - Entry is a conference abstract, a statement, an editorial/introduction or a non-scientific journal article  - Entry is an Erratum of an original scientific article |
| Setting | - hospital or a specific hospital ward (i.e. maternity ward, emergency department, …)  - GP practices, ambulatory care centres, mental health centres, pharmacies  - nursing homes  - or: health care system | - laboratories  - dental care / oral health  - military health care |
| Content |  | - focus on other topic than health care (education, environment)  - focus on health insurance efficiency  - focus on other type of efficiency than economic efficiency (treatment efficiency, energy efficiency, training efficiency, imaging efficiency, …)  - focusing on (patients with) a specific disease/condition, screening, intervention or medication |

# Screening guide for the full-text screening (additional criteria in *italics*):

| **Criterion** | **Include** | **Exclude** |
| --- | --- | --- |
| *Availability* | *- Full text is available* | *- Full text is not available* |
| Language | - Full text is in English, German or French | - Full text is not English, German or French |
| Publication type | - Full text is an original scientific article  - Full text is a book chapter | - Full text is a whole book  - Full text is a systematic review or other type of review  - Full text is a conference abstract, a statement, an editorial/introduction or a non-scientific journal article  - Full text is an Erratum of an original scientific article |
| Setting | - hospital or a specific hospital ward (i.e. maternity ward, emergency department, hospital pharmacy …)  - GP practices, ambulatory care centres, mental health centres, pharmacies  - nursing homes  - or: health care system | - laboratories  - dental care / oral health  - military health care  *- operating room (if focus only on one specific room, not on the chirurgical ward/all ORs of a hospital)* |
| Content | - focus on health care or specific health care providers | - focus on other topic than health care (education, environment)  - focus on health insurance efficiency  - focus on other type of efficiency than economic efficiency (treatment efficiency, energy efficiency, training efficiency, imaging efficiency, *…)*  - focusing on (patients with) a specific disease/condition, screening, intervention or medication  *- focusing purely on technical aspects of methodology* |
| *Definition* | *- contains one or multiple of the following:*  *(proposed) definition* ***or*** *(proposed) measurement of “efficiency” or “inefficiency”* | *- contains no explicit or implicit definition* ***or*** *measurement (proposition) of efficiency/inefficiency*  *🡪 when efficiency/efficient/… is only used as a descriptive word without further explanation* |

Please report the reason for exclusion (availability, language, publication type, setting, content, definition) for each source.
