# Supplementary material for: Definition and understanding of “efficiency” in healthcare provision research: a scoping review
Source: Front Public Health. 2024 Nov 4;12:1439788. doi: 10.3389/fpubh.2024.1439788 (PMC11571063; doi:10.3389/fpubh.2024.1439788)
Supplement: Supplementary Table 4 — Full reference list. [file Table_4.docx]

Supplementary Material 4: Full reference list

All 389 studies included in the review, presented in alphabetical order:

| **#** | **Reference** |
| --- | --- |
|  | Abeney, A., & Yu, K. A. M. (2015). Measuring the Efficiency of the Canadian Health Care System. *Canadian Public Policy*, *41*(4), 320-331. https://doi.org/10.3138/cpp.2013-044 |
|  | Aboagye, A. Q. Q., & Degboe, A. N. K. (2011). Cost analysis and efficiency of sub-district health facilities in two districts in Ghana. *The International journal of health planning and management*, *26*(2), 173-190. https://doi.org/10.1002/hpm.1047 |
|  | Abolghasem, S., Toloo, M., & Amézquita, S. (2019). Cross-efficiency evaluation in the presence of flexible measures with an application to healthcare systems. *Health care management science*, *22*(3), 512-533. https://doi.org/10.1007/s10729-019-09478-0 |
|  | Afzali, H. H. A., Moss, J. R., & Mahmood, M. A. (2009). A conceptual framework for selecting the most appropriate variables for measuring hospital efficiency with a focus on Iranian public hospitals. *Health services management research*, *22*(2), 81- 91. https://doi.org/10.1258/hsmr.2008.008020 |
|  | Ahmed, S., Hasan, M. Z., Laokri, S., Jannat, Z., Ahmed, M. W., Dorin, F., Vargas, V., & Khan, J. A. M. (2019). Technical efficiency of public district hospitals in Bangladesh: a data envelopment analysis. *Cost effectiveness and resource allocation: C/E*, *17*, 15. https://doi.org/10.1186/s12962-019-0183-6 |
|  | Ahmed, S., Hasan, M. Z., MacLennan, M., Dorin, F., Ahmed, M. W., Hasan, M. M., Hasan, S. M., Islam, M. T., & Khan, J. A. M. (2019). Measuring the efficiency of health systems in Asia: a data envelopment analysis. *BMJ open*, *9*(3), e022155. https://doi.org/10.1136/bmjopen-2018-022155 |
|  | Ajlouni, M. d. M., Zyoud, A., Jaber, B., Shaheen, H., Al-Natour, M., & Anshasi, R. J. (2013). The Relative Efficiency of Jordanian Public Hospitals Using Data Envelopment Analysis and Pabon Lasso Diagram. *Global Journal of Business Research*, *7*(2), 59-72. https://doi.org/http://www.theibfr.com/gjbrsample.htm |
|  | Akazili, J., Adjuik, M., Jehu-Appiah, C., & Zere, E. (2008). Using data envelopment analysis to measure the extent of technical efficiency of public health centres in Ghana. *BMC international health and human rights*, *8*, 11.  https://doi.org/10.1186/1472-698X-8-11 |
|  | Al-Amin, M., Makarem, S. C., & Rosko, M. (2016). Efficiency and hospital effectiveness in improving Hospital Consumer Assessment of Healthcare Providers and Systems ratings. *Health care management review*, *41*(4), 296-305. https://doi.org/10.1097/HMR.0000000000000076 |
|  | Alatawi, A. D., Niessen, L. W., & Khan, J. A. M. (2020). Determinants of Technical Efficiency in Public Hospitals: The Case of Saudi Arabia. *Health economics review*, *10*(1), 25. https://doi.org/10.1186/s13561-020-00282-z |
|  | Alatawi, A. D., Niessen, L. W., & Khan, J. A. M. (2020). Efficiency evaluation of public hospitals in Saudi Arabia: an application of data envelopment analysis. *BMJ open*, *10*(1), e031924. https://doi.org/10.1136/bmjopen-2019-031924 |

|  | Alhassan, R. K., Nketiah-Amponsah, E., Akazili, J., Spieker, N., Arhinful, D. K., & Rinke de Wit, T. F. (2015). Efficiency of private and public primary health facilities accredited by the National Health Insurance Authority in Ghana [Article]. *Cost Effectiveness and Resource Allocation*, *13*(1).  https://doi.org/10.1186/s12962-015-0050-z |
| --- | --- |
|  | Allin, S., Grignon, M., & Wang, L. (2016). The determinants of efficiency in the Canadian health care system. *Health economics, policy, and law*, *11*(1), 39-65. https://doi.org/10.1017/S1744133115000274 |
|  | Allin, S., Veillard, J., Wang, L., & Grignon, M. (2015). How Can Health System Efficiency Be Improved in Canada? *Healthcare policy = Politiques de sante*, *11*(1), 33-45. |
|  | Aloh, H. E., Onwujekwe, O. E., Aloh, O. G., & Nweke, C. J. (2020). Is bed turnover rate a good metric for hospital scale efficiency? A measure of resource utilization rate for hospitals in Southeast Nigeria. *Cost effectiveness and resource allocation : C/E*, *18*, 21. https://doi.org/10.1186/s12962-020-00216-w |
|  | Alonso, J. M., Clifton, J., & Díaz-Fuentes, D. (2015). The impact of New Public Management on efficiency: an analysis of Madrid's hospitals. *Health policy (Amsterdam, Netherlands)*, *119*(3), 333-340. https://doi.org/10.1016/j.healthpol.2014.12.001 |
|  | Al-Shammari, M. (1999). A multi-criteria data envelopment analysis model for measuring the productive efficiency of hospitals [Article]. *International Journal of Operations & Production Management*, *19*(9/10), 879-890. https://doi.org/10.1108/01443579910280205 |
|  | Alwaked, A. A., Al‐qalawi, U. R., & Azaizeh, S. Y. (2020). Efficiency of Jordanian public hospitals (2006–2015) [Article]. *Journal of Public Affairs (14723891)*, 1. https://doi.org/10.1002/pa.2383 |
|  | Amado, C. A. d. E. F., & Santos, S. P. D. (2009). Challenges for performance assessment and improvement in primary health care: the case of the Portuguese health centres. *Health policy (Amsterdam, Netherlands)*, *91*(1), 43-56. https://doi.org/10.1016/j.healthpol.2008.11.008 |
|  | Anastasios, T., Miltiadis, N., Styliani, T., & Panagiotis, P. (2021). Measuring Technical Efficiency of Health Centers in Greece: A Data Envelopment Analysis Application for the Primary Health System of Greece. *European Research Studies*, *24*, 1333- 1353. https://doi.org/https://www.ersj.eu/index.php |
|  | Ancarani, A., Ayach, A., Di Mauro, C., Gitto, S., & Mancuso, P. (2016). Does Religious Diversity in Health Team Composition Affect Efficiency? Evidence from Dubai [Article]. *British Journal of Management*, *27*(4), 740-759. https://doi.org/10.1111/1467-8551.12184 |
|  | Ancarani, A., Di Mauro, C., & Giammanco, M. D. (2009). The impact of managerial and organizational aspects on hospital wards’ efficiency: Evidence from a case study [Article]. *European Journal of Operational Research*, *194*(1), 280-293. https://doi.org/10.1016/j.ejor.2007.11.046 |
|  | Andrews, A. (2021). An application of PCA-DEA with the double-bootstrap approach to estimate the technical efficiency of New Zealand District Health Boards. *Health economics, policy, and law*, 1-25. https://doi.org/10.1017/S1744133120000420 |
|  | Andrews, A. (2021). The Efficiency of New Zealand District Health Boards in Administrating Public Funds: An Application of Bootstrap DEA and Beta Regression [Article]. *International Journal of Public Administration*, *44*(14), 1297- 1308. https://doi.org/10.1080/01900692.2020.1755685 |
|  | Andrews, A., Temoso, O., & Kimpton, S. (2021). Persistent and Transient Inefficiency of Australian States and Territories in Providing Public Hospital Services: An Application of Bayesian Stochastic Finite Mixture Frontier Analysis [Article]. *Economic Papers*, *40*(2), 104-115. https://doi.org/10.1111/1759-3441.12310 |
|  | Androutsou, L., Geitona, M., & Yfantopoulos, J. (2011). Measuring Efficiency and Productivity Across Hospitals in the Regional Health Authority of Thessaly, in Greece. *Journal of Health Management*, *13*(2), 121-140. https://doi.org/10.1177/097206341101300201 |
|  | Anton, S. G. (2013). Technical Efficiency in the Use of Health Care Resources: A Cross- Country Analysis. *Scientific Annals of the 'Alexandru Ioan Cuza' University of Iasi*, *60*(1), 31-42. |
|  | Applanaidu, S.-D., Samsudin, S., Ali, J., Dash, U., & Chik, A. R. (2014). Technical and Scale Efficiency of Public District Hospitals in Kedah, Malaysia: A Data Envelopment Analysis (DEA). *Journal of Health Management*, *16*(3), 327-335. https://doi.org/10.1177/0972063414539595 |
|  | Araújo, C., Barros, C. P., & Wanke, P. (2014). Efficiency determinants and capacity issues in Brazilian for-profit hospitals. *Health care management science*, *17*(2), 126-138. https://doi.org/10.1007/s10729-013-9249-8 |
|  | Asghar, N., Ali, M., Farooq, F., & Talpur, U. (2019). Delivery of Health Care Service in the Organization of Islamic Cooperation (OIC) Member States and Regional Diversity: A Bootstrap DEA Analysis. *Review of Economics and Development Studies*, *5*(1), 59-66. |
|  | Athanassopoulos, A. D., Gounaris, C., & Sissouras, A. (1999). A descriptive assessment of the production and cost efficiency of general hospitals in Greece. *Health care management science*, *2*(2), 97-106. https://doi.org/10.1023/a:1019023408924 |
|  | Atılgan, E. (2016). The Technical Efficiency of Hospital Inpatient Care Services: An Application for Turkish Public Hospitals [Article]. *Business & Economics Research Journal*, *7*(2), 203-214. https://doi.org/10.20409/berj.2016217537 |
|  | Au, N., Hollingsworth, B., & Spinks, J. (2014). Measuring the Efficiency of Health Services in Lower-Income Countries: The Case of Papua New Guinea. *Development Policy Review*, *32*(2), 259-272. |
|  | Aung, Y. N., Nur, A. M., Ismail, A., & Aljunid, S. M. (2020). Determining the Cost and Length of Stay at Intensive Care Units and the Factors Influencing Them in a Teaching Hospital in Malaysia. *Value in health regional issues*, *21*, 149-156. https://doi.org/10.1016/j.vhri.2019.09.006 |
|  | Ayiko, R., Mujasi, P. N., Abaliwano, J., Turyareeba, D., Enyaku, R., Anguyo, R., Odoch, W., Bakibinga, P., & Aliti, T. (2020). Levels, trends and determinants of technical efficiency of general hospitals in Uganda: data envelopment analysis and Tobit regression analysis. *BMC health services research*, *20*(1), 916. https://doi.org/10.1186/s12913-020-05746-w |
|  | Bağci, H., & Konca, M. (2021). Evaluating the Technical Efficiency of Hospitals Providing Tertiary Health Care in Turkey: An Application Based on Data Envelopment Analysis. *Hospital topics*, *99*(2), 49-63. https://doi.org/10.1080/00185868.2020.1830008 |
|  | Barasa, E., Musiega, A., Hanson, K., Nyawira, L., Mulwa, A., Molyneux, S., Maina, I., Tsofa, B., Normand, C., & Jemutai, J. (2021). Level and determinants of county health system technical efficiency in Kenya: two stage data envelopment analysis [Article]. *Cost Effectiveness and Resource Allocation*, *19*(1).  https://doi.org/10.1186/s12962-021- 00332-1 |
|  | Bardey, D., & Pichetti, S. (2004). Estimation de l'efficience des depenses de sante au niveau departemental par la methode DEA. (Estimation of the Efficiency of Health Expenditure in the French Departements Using the DEA Method. With English summary.). *Economie et Prevision*(166), 59-69. |
|  | Barnum, D. T., Shields, K. L., Walton, S. M., & Schumock, G. T. (2011). Improving the efficiency of distributive and clinical services in hospital pharmacy. *Journal of medical systems*, *35*(1), 59-70. https://doi.org/10.1007/s10916-009-9341-2 |
|  | Barpanda, S., & Sreekumar, N. (2020). Performance Analysis of Hospitals in Kerala Using Data Envelopment Analysis Model. *Journal of Health Management*, *22*(1), 25-40. https://doi.org/10.1177/0972063420908372 |
|  | Barros, C., de Menezes, A., & Vieira, J. (2013). Measurement of hospital efficiency, using a latent class stochastic frontier model [Article]. *Applied Economics*, *45*(1), 47-54. https://doi.org/10.1080/00036846.2011.579061 |
|  | Bashir, S., & Nasir, M. (2020). Tradeoff between efficiency and perceived quality: evidence from patient-level data. *International journal for quality in health care : journal of the International Society for Quality in Health Care*, *32*(9), 591-598. https://doi.org/10.1093/intqhc/mzaa098 |
|  | Becker, E. R., & Potter, S. J. (2002). Organizational rationality, performance, and social responsibility: results from the hospital industry. *Journal of health care finance*, *29*(1), 23-48. |
|  | Beech, R., & Larkinson, J. (1990). Estimating the financial savings from maintaining the level of acute services with fewer hospital beds. *The International journal of health planning and management*, *5*(2), 89-103. https://doi.org/10.1002/hpm.4740050203 |
|  | Behr, A., & Theune, K. (2017). Health System Efficiency: A Fragmented Picture Based on OECD Data. *PharmacoEconomics - open*, *1*(3), 203-221. https://doi.org/10.1007/s41669-017-0010-y |
|  | BekaroĞLu, C., & Heffley, D. (2018). A MULTI-STAGE EFFICIENCY ANALYSIS OF OECD HEALTHCARE SYSTEMS [Article]. *OECD SAĞLIK SİSTEMLERİ ÇOK SAFHALI ETKİNLİK ANALİZİ.*, *16*(2), 264-285. https://doi.org/10.11611/yead.421180 |
|  | Benčina, J., Devjak, S., & Umek, L. (2014). Determining the Adequacy of Operation of DMUs in Health Care [Article]. *Management (1820-0222)*, *19*(73), 25-34. https://doi.org/10.7595/management.fon.2014.0031 |
|  | Bernet, P. M., Moises, J., & Valdmanis, V. G. (2011). Social efficiency of hospital care delivery: frontier analysis from the consumer's perspective. *Medical care research and review : MCRR*, *68*(1 Suppl), 36S-54S. https://doi.org/10.1177/1077558710366267 |
|  | Björkgren, M. A., Häkkinen, U., & Linna, M. (2001). Measuring efficiency of long-term care units in Finland. *Health care management science*, *4*(3), 193-200. https://doi.org/10.1023/a:1011444815466 |
|  | Blank, J. L. T., & Valdmanis, V. G. (2010). Environmental factors and productivity on Dutch hospitals: a semi-parametric approach. *Health care management science*, *13*(1), 27-34. https://doi.org/10.1007/s10729-009-9104-0 |
|  | Blatnik, P., Bojnec, Š., & Tušak, M. (2017). Measuring Efficiency of Secondary Healthcare Providers in Slovenia. *Open medicine (Warsaw, Poland)*, *12*, 214-225. https://doi.org/10.1515/med-2017-0031 |
|  | Blöndal, B., & Ásgeirsdóttir, T. L. (2019). Costs and efficiency of gatekeeping under varying numbers of general practitioners. *The International journal of health planning and management*, *34*(1), 140-156. https://doi.org/10.1002/hpm.2601 |
|  | Bobo, F. T., Woldie, M., Wordofa, M. A., Tsega, G., Agago, T. A., Wolde-Michael, K., Ibrahim, N., & Yesuf, E. A. (2018). Technical efficiency of public health centers in three districts in Ethiopia: two-stage data envelopment analysis [Article]. *BMC research notes*, *11*(1), 465. https://doi.org/10.1186/s13104-018-3580-6 |
|  | Bonasia, M., Kounetas, K., & Oreste, N. (2020). Assessment of regional productive performance of European health systems under a metatechnology framework [Article]. *Economic Modelling*, *84*, 234-248. https://doi.org/10.1016/j.econmod.2019.04.013 |
|  | Borisov, D., Cicea, C., & Turlea, C. (2012). DEA MODEL FOR ASSESSING EFFICIENCY IN PROVIDING HEALTH CARE AND MANAGEMENT DECISIONS [Article]. *Management Research & Practice*, *4*(1), 5-18. |
|  | Boronat, F., Budia, A., Broseta, E., Ruiz-Cerdá, J. L., & Vivas-Consuelo, D. (2018). Application of Lean Healthcare methodology in a urology department of a tertiary hospital as a tool for improving efficiency. *Actas urologicas espanolas*, *42*(1), 42-48. https://doi.org/10.1016/j.acuro.2017.03.009 |
|  | Brownell, M. D., & Roos, N. P. (1995). Variation in length of stay as a measure of efficiency in Manitoba hospitals. *CMAJ : Canadian Medical Association journal = journal de l'Association medicale canadienne*, *152*(5), 675-682. |
|  | Burgess, J. F., & Wilson, P. W. (1998). VARIATION IN INEFFICIENCY AMONG US HOSPITALS [Article]. *INFOR*, *36*(3), 84-102. https://doi.org/10.1080/03155986.1998.11732348 |
|  | Burgess Jr, J. F., & Wilson, P. W. (1995). Decomposing Hospital Productivity Changes, 1985-1988: A Nonparametric Malmquist Approach [Article]. *Journal of Productivity Analysis*, *6*(4), 343-363. https://doi.org/10.1007/BF01073525 |
|  | Burgess Jr, J. F., & Wilson, P. W. (1996). Hospital Ownership and Technical Inefficiency [Article]. *Management Science*, *42*(1), 110-123. https://doi.org/10.1287/mnsc.42.1.110 |
|  | Burney, N. A., Mohammad, O. E., & Al-Ramadhan, M. A. (1999). Assessing the cost of inefficiencies: The case of the public health care system in Kuwait [Article]. *International Social Science Review*, *74*(1/2), 20. |
|  | Campanella, P., Azzolini, E., Izzi, A., Pelone, F., De Meo, C., La Milia, D., Specchia, M. L., & Ricciardi, W. (2017). Hospital efficiency: how to spend less maintaining quality? *Annali dell'Istituto superiore di sanita*, *53*(1), 46-53. https://doi.org/10.4415/ANN_17_01_10 |
|  | Campos, M. S., Fernández-Montes, A., Gavilan, J. M., & Velasco, F. (2016). Public resource usage in health systems: a data envelopment analysis of the efficiency of health systems of autonomous communities in Spain. *Public health*, *138*, 33-40. https://doi.org/10.1016/j.puhe.2016.03.003 |
|  | Carrillo, M., & Jorge, J. (2017). DEA-Like Efficiency Ranking of Regional Health Systems in Spain [Article]. *Social indicators research*, *133*(3), 1133-1149. https://doi.org/10.1007/s11205-016-1398-y |
|  | Castelli, A., Street, A., Verzulli, R., & Ward, P. (2015). Examining variations in hospital productivity in the English NHS. *The European journal of health economics : HEPAC : health economics in prevention and care*, *16*(3), 243-254. https://doi.org/10.1007/s10198-014-0569-5 |
|  | Cavalieri, M., Guccio, C., Lisi, D., & Pignataro, G. (2018). Does the Extent of per Case Payment System Affect Hospital Efficiency? [Article]. *Public Finance Review*, *46*(1), 117-149. https://doi.org/10.1177/1091142116651487 |
|  | Çelik, Y., Khan, M., & Hikmet, N. (2017). Achieving value for money in health: a comparative analysis of OECD countries and regional countries. *The International journal of health planning and management*, *32*(4), e279-e298. https://doi.org/10.1002/hpm.2375 |
|  | Cellini, R., Pignataro, G., & Rizzo, I. (2000). Competition and Efficiency in Health Care: An Analysis of the Italian Case. *International Tax and Public Finance*, *7*(4-5), 503-519. |
|  | Chang, H.-h. (1998). Determinants of hospital efficiency: The case of central [Article]. *Omega*, *26*(2), 307. https://doi.org/10.1016/S0305-0483(98)00014-0 |
|  | Chen, A., Kim, E. A., Aigner, D. J., Afifi, A., & Caprioli, J. (2015). Index to Estimate the Efficiency of an Ophthalmic Practice. *JAMA ophthalmology*, *133*(8), 924-929. https://doi.org/10.1001/jamaophthalmol.2015.1447 |
|  | Chen, Y., Wang, J., Zhu, J., Sherman, H. D., & Chou, S.-Y. (2019). How the Great Recession affects performance: a case of Pennsylvania hospitals using DEA [Article]. *Annals of Operations Research*, *278*(1/2), 77-99. https://doi.org/10.1007/s10479-017-2516-1 |
|  | Chen, Z., Chen, X., Gan, X., Bai, K., Baležentis, T., & Cui, L. (2020). Technical Efficiency of Regional Public Hospitals in China Based on the Three-Stage DEA. *International journal of environmental research and public health*, *17*(24). https://doi.org/10.3390/ijerph17249383 |
|  | Cheng, G., & Zervopoulos, P. D. (2014). Estimating the technical efficiency of health care systems: A cross-country comparison using the directional distance function [Article]. *European Journal of Operational Research*, *238*(3), 899-910. https://doi.org/10.1016/j.ejor.2014.05.007 |
|  | Choi, J. H., Fortsch, S. M., Park, I., & Jung, I. (2017). Efficiency of U.S. hospitals between 2001 and 2011 [Article]. *Managerial & Decision Economics*, *38*(8), 1071-1081. https://doi.org/10.1002/mde.2846 |
|  | Choi, J. H., Park, I., Jung, I., & Dey, A. (2017). Complementary effect of patient volume and quality of care on hospital cost efficiency. *Health care management science*, *20*(2), 221-231. https://doi.org/10.1007/s10729-015-9348-9 |
|  | Chu, C.-L., Chiang, T.-L., & Chang, R.-E. (2011). Hospital competition and inpatient services efficiency in Taiwan: a longitudinal study. *Health economics*, *20*(10), 1268- 1280. https://doi.org/10.1002/hec.1676 |
|  | Chu, H.-L., Wang, C.-C., & Shiu, S. F. (2009). Effect of participating in Taiwan Quality Indicator Project on hospital efficiency in Taiwan. *Journal of health care finance*, *35*(4), 32-41. |
|  | Chua, C. L., Palangkaraya, A., & Yong, J. (2011). Hospital Competition, Technical Efficiency and Quality [Article]. *Economic Record*, *87*(277), 252-268. https://doi.org/10.1111/j.1475-4932.2010.00704.x |
|  | Cinaroglu, S. (2020). Integrated k-means clustering with data envelopment analysis of public hospital efficiency. *Health care management science*, *23*(3), 325-338. https://doi.org/10.1007/s10729-019-09491-3 |
|  | Cinaroglu, S. (2021). Changes in hospital efficiency and size: An integrated propensity score matching with data envelopment analysis [Article]. *Socio-economic planning sciences*, *76*, N.PAG-N.PAG. https://doi.org/10.1016/j.seps.2020.100960 |
|  | Cinaroglu, S. (2021). Efficiency in health services based on professionals consensus quality indicators [Article]. *International Journal of Healthcare Management*, *14*(4), 993- 1001. https://doi.org/10.1080/20479700.2020.1724436 |
|  | Cinaroglu, S. (2021). Intensive Care Unit Services Preparedness for the Pandemic: An Efficiency Analysis...47th annual meeting (online) of the EURO Working Group on Operational Research Applied to Health Services: July 5-9, 2021. *Health services insights*, 1-9. https://doi.org/10.1177/11786329211037527 |
|  | Cordero Ferrera, J. M., Crespo Cebada, E., & Murillo Zamorano, L. R. (2014). The effect of quality and socio-demographic variables on efficiency measures in primary health care. *The European journal of health economics : HEPAC : health economics in prevention and care*, *15*(3), 289-302. https://doi.org/10.1007/s10198-013-0476-1 |
|  | Cordero, J. M., García-García, A., Lau-Cortés, E., & Polo, C. (2021). Efficiency and Productivity Change of Public Hospitals in Panama: Do Management Schemes Matter? *International journal of environmental research and public health*, *18*(16). https://doi.org/10.3390/ijerph18168630 |
|  | Cordero, J. M., García‐García, A., Lau‐Cortés, E., & Polo, C. (2021). Assessing Panamanian hospitals' performance with alternative frontier methods [Article]. *International Transactions in Operational Research*, 1. https://doi.org/10.1111/itor.13013 |
|  | Cox, J. A., Bernard, A. C., Bottiggi, A. J., Chang, P. K., Talley, C. L., Tucker, B., Davenport, D. L., & Kearney, P. A. (2014). Influence of in-house attending presence on trauma outcomes and hospital efficiency. *Journal of the American College of Surgeons*, *218*(4), 734-738. https://doi.org/10.1016/j.jamcollsurg.2013.12.027 |
|  | Coyne, J. S., Richards, M. T., Short, R., Shultz, K., & Singh, S. G. (2009). Hospital cost and efficiency: do hospital size and ownership type really matter? *Journal of healthcare management / American College of Healthcare Executives*, *54*(3), 163-174. |
|  | Daidone, S., & D'Amico, F. (2009). Technical efficiency, specialization and ownership form: evidences from a pooling of Italian hospitals [Article]. *Journal of Productivity Analysis*, *32*(3), 203-216. https://doi.org/10.1007/s11123-009-0137-7 |
|  | Dalmau-Matarrodona, E., & Jaume, P.-J. (1998). Market Structure and Hospital Efficiency: Evaluating Potential Effects of Deregulation in a National Health Service [Article]. *Review of Industrial Organization*, *13*(4), 447-466. |
|  | Danilov, A. V. (2021). Scenarios for restructuring the regional inpatient healthcare system based on efficiency indicators and specialization indices [Article]. *International Journal of Healthcare Management*, 1-8. https://doi.org/10.1080/20479700.2020.1870366 |
|  | Davey, S., Raghav, S. K., Singh, J. V., Davey, A., & Singh, N. (2015). A Comparative Evaluation of Public Health Centers with Private Health Training Centers on Primary Healthcare Parameters in India: a Study by Data Envelopment Analysis Technique. *Indian journal of community medicine : official publication of Indian Association of Preventive & Social Medicine*, *40*(4), 252-257.  https://doi.org/10.4103/0970-0218.164394 |
|  | de Cos, P. H., & Moral-Benito, E. (2014). Determinants of health-system efficiency: evidence from OECD countries. *International journal of health care finance and economics*, *14*(1), 69-93. https://doi.org/10.1007/s10754-013-9140-7 |
|  | De Nicola, A., Gitto, S., Mancuso, P., & Valdmanis, V. (2014). Healthcare reform in Italy: an analysis of efficiency based on nonparametric methods. *The International journal of health planning and management*, *29*(1), e48-e63. https://doi.org/10.1002/hpm.2183 |
|  | de Sousa, K. M., Pinhanez, M. D. M. S. F., do Monte, P. A., & Diniz, J. A. (2020). Salary, Financial Autonomy and Efficiency of Healthcare Systems in Local Governments. *Applied Economics Letters*, *27*(2), 122-126. https://doi.org/http://www.tandfonline.com/loi/rael20 |
|  | Deidda, M., Lupiáñez-Villanueva, F., Codagnone, C., & Maghiros, I. (2014). Using data envelopment analysis to analyse the efficiency of primary care units. *Journal of medical systems*, *38*(10), 122. https://doi.org/10.1007/s10916-014-0122-1 |
|  | DeLaney, M., Zimmerman, K. D., Strout, T. D., & Fix, M. L. (2013). The effect of medical students and residents on measures of efficiency and timeliness in an academic medical center emergency department. *Academic medicine : journal of the Association of American Medical Colleges*, *88*(11), 1723-1731. https://doi.org/10.1097/ACM.0b013e3182a7f1f8 |
|  | DePuccio, M. J., & Ozcan, Y. A. (2017). Exploring efficiency differences between medical home and non-medical home hospitals [Article]. *International Journal of Healthcare Management*, *10*(3), 147-153. https://doi.org/10.1080/20479700.2015.1101913 |
|  | Ding, J., Hu, X., Zhang, X., Shang, L., Yu, M., & Chen, H. (2018). Equity and efficiency of medical service systems at the provincial level of China's mainland: a comparative study from 2009 to 2014. *BMC public health*, *18*(1), 214. https://doi.org/10.1186/s12889-018-5084-7 |
|  | Dittman, D. A., Capettini, R., & Morey, R. C. (1991). Measuring efficiency in acute care hospitals: an application of data envelopment analysis. *Journal of health and human resources administration*, *14*(1), 89-108. |
|  | Dormont, B., & Milcent, C. (2012). Comment evaluer la productivite et l'efficacite des hopitaux publics et prives? Les enjeux de la convergence tarifaire. (How Should the Productivity and Efficiency of Public and Private Hospitals Be Assessed? Issues at Stake in Price Convergence. With English summary.). *Economie et Statistique*(455- 456), 143-173. |
|  | Eklom, B., Tracy, S., & Callander, E. (2021). An exploration of potential output measures to assess efficiency and productivity for labour and birth in Australia. *BMC pregnancy and childbirth*, *21*(1), 703. https://doi.org/10.1186/s12884-021-04181-x |
|  | Evans, D. B., Tandon, A., Murray, C. J. L., & Lauer, J. A. (2001). Comparative efficiency of national health systems: Cross national econometric analysis [Article]. *British medical journal*, *323*(7308), 307-310. https://doi.org/10.1136/bmj.323.7308.307 |
|  | Falavigna, G., Ippoliti, R., & Manello, A. (2013). Hospital organization and performance: a directional distance function approach. *Health care management science*, *16*(2), 139-151. https://doi.org/10.1007/s10729-012-9217-8 |
|  | Färe, R., Grosskopf, S., Lindgren, B., & Poullier, J. P. (1997). Productivity growth in health- care delivery. *Medical care*, *35*(4), 354-366.  https://doi.org/10.1097/00005650-199704000-00006 |
|  | Färe, R., Grosskopf, S., & Valdaunis, V. (1989). Capacity, Competiton and Efficiency in Hospitals: A Nonparametric Approach [Article]. *Journal of Productivity Analysis*, *1*(2), 123-138. |
|  | Farsi, M. (2008). The temporal variation of cost-efficiency in Switzerland’s hospitals: an application of mixed models [Article]. *Journal of Productivity Analysis*, *30*(2), 155- 168. https://doi.org/10.1007/s11123-008-0105-7 |
|  | Ferreira, C., Marques, R. C., & Nicola, P. (2013). On evaluating health centers groups in Lisbon and Tagus Valley: efficiency, equity and quality. *BMC health services research*, *13*, 529. https://doi.org/10.1186/1472-6963-13-529 |
|  | Ferreira, D. C., & Nunes, A. M. (2019). Technical efficiency of Portuguese public hospitals: A comparative analysis across the five regions of Portugal. *The International journal of health planning and management*, *34*(1), e411-e422. https://doi.org/10.1002/hpm.2658 |
|  | Ferreira, D. C., Nunes, A. M., & Marques, R. C. (2018). Doctors, nurses, and the optimal scale size in the Portuguese public hospitals. *Health policy (Amsterdam, Netherlands)*, *122*(10), 1093-1100. https://doi.org/10.1016/j.healthpol.2018.06.009 |
|  | Ferreira, D. C., Nunes, A. M., & Marques, R. C. (2020). Operational efficiency vs clinical safety, care appropriateness, timeliness, and access to health care: The case of Portuguese public hospitals [Article]. *Journal of Productivity Analysis*, *53*(3), 355- 375. https://doi.org/10.1007/s11123-020-00578-6 |
|  | Fixler, T., Paradi, J. C., & Yang, X. (2014). A data envelopment analysis approach for measuring the efficiency of Canadian acute care hospitals [Article]. *Health services management research*, *27*(3-4), 57-69. https://doi.org/10.1177/0951484815601876 |
|  | Flokou, A., Aletras, V., & Niakas, D. (2017). A window-DEA based efficiency evaluation of the public hospital sector in Greece during the 5-year economic crisis. *PloS one*, *12*(5), e0177946. https://doi.org/10.1371/journal.pone.0177946 |
|  | Forbes, I., Hindle, D., Degeling, P., Zhang, K., Xu, L., Meng, Q., & Wang, J. (2002). The effects of increased market competition on hospital services in Shandong and Henan Provinces. *Australian health review : a publication of the Australian Hospital Association*, *25*(2), 52-65. https://doi.org/10.1071/ah020052 |
|  | Franco Miguel, J. L., Fullana Belda, C., & Rúa Vieites, A. (2019). Analysis of the technical efficiency of the forms of hospital management based on public-private collaboration of the Madrid Health Service, as compared with traditional management. *The International journal of health planning and management*, *34*(1), 414-442. https://doi.org/10.1002/hpm.2678 |
|  | Frech, T. E. (1998). *Efficiency, Growth and Concentration: An Empirical Analysis of Hospital Markets*. Department of Economics, UC Santa Barbara, University of California at Santa Barbara, Economics Working Paper Series. |
|  | Friesner, D., Roseman, R., & McPherson, M. Q. (2008). Are hospitals seasonally inefficient? Evidence from Washington State [Article]. *Applied Economics*, *40*(6), 699-723. https://doi.org/10.1080/00036840600749730 |
|  | Gai, R. Y., Zhou, C. C., Xu, L. Z., Zhu, M., Wang, X. Z., Li, S. X., Zheng, W. G., Song, P. P., Yang, X. L., Fang, L. Y., Zhen, Y. C., & Tang, W. (2010). Health resource allocation and productive efficiency of Chinese county hospitals: data from 1993 to 2005. *Bioscience trends*, *4*(5), 218-224. |
|  | Gannon, B. (2005). Testing for Variation in Technical Efficiency of Hospitals in Ireland. *Economic and Social Review*, *36*(3), 273-294. https://doi.org/http://www.esr.ie/issue/archive |
|  | Gao, Q., & Wang, D. (2021). Hospital efficiency and equity in health care delivery: A study based in China [Article]. *Socio-economic planning sciences*, *76*, N.PAG-N.PAG. https://doi.org/10.1016/j.seps.2020.100964 |
|  | Garavaglia, G., Lettieri, E., Agasisti, T., & Lopez, S. (2011). Efficiency and quality of care in nursing homes: an Italian case study. *Health care management science*, *14*(1), 22- 35. https://doi.org/10.1007/s10729-010-9139-2 |
|  | García-Romero, A., Escribano, Á., & Tribó, J. A. (2017). The impact of health research on length of stay in Spanish public hospitals [Article]. *Research Policy*, *46*(3), 591-604. https://doi.org/10.1016/j.respol.2017.01.006 |
|  | Gautam, S., Hicks, L., Johnson, T., & Mishra, B. (2013). Measuring the performance of Critical Access Hospitals in Missouri using data envelopment analysis. *The Journal of rural health : official journal of the American Rural Health Association and the National Rural Health Care Association*, *29*(2), 150-158.  https://doi.org/10.1111/j.1748-0361.2012.00439.x |
|  | Gavurova, B., Kocisova, K., & Sopko, J. (2021). Health system efficiency in OECD countries: dynamic network DEA approach. *Health economics review*, *11*(1), 40. https://doi.org/10.1186/s13561-021-00337-9 |
|  | Geitona, M., Androutsou, L., Kotsopoulos, N., & Gourgoulianis, K. (2014). Measuring the efficiency among secondary and university pulmonary hospital clinics in Greece [Article]. *Pneumon*, *27*(1), 31-36. |
|  | Giuffrida, A. (1999). Productivity and efficiency changes in primary care: a Malmquist index approach. *Health care management science*, *2*(1), 11-26. https://doi.org/10.1023/a:1019067223945 |
|  | Gok, M. S., & Altındağ, E. (2015). Analysis of the cost and efficiency relationship: experience in the Turkish pay for performance system. *The European journal of health economics : HEPAC : health economics in prevention and care*, *16*(5), 459- 469. https://doi.org/10.1007/s10198-014-0584-6 |
|  | Gok, M. S., & Sezen, B. (2013). Analyzing the ambiguous relationship between efficiency, quality and patient satisfaction in healthcare services: the case of public hospitals in Turkey. *Health policy (Amsterdam, Netherlands)*, *111*(3), 290-300. https://doi.org/10.1016/j.healthpol.2013.05.010 |
|  | González, E., Cárcaba, A., & Ventura, J. (2010). Value efficiency analysis of health systems: Does public financing play a role? [Article]. *Journal of Public Health*, *18*(4), 337- 350. https://doi.org/10.1007/s10389-009-0311-4 |
|  | Grosskopf, S., Margaritis, D., & Valdmanis, V. (2004). Competitive effects on teaching hospitals [Article]. *European Journal of Operational Research*, *154*(2), 515. https://doi.org/10.1016/S0377-2217(03)00185-1 |
|  | Grosskopf, S., & Valdmanis, V. (1987). Measuring hospital performance. A non-parametric approach. *Journal of health economics*, *6*(2), 89-107.  https://doi.org/10.1016/0167-6296(87)90001-4 |
|  | Guerrini, A., Romano, G., Campedelli, B., Moggi, S., & Leardini, C. (2018). Public vs. Private in Hospital Efficiency: Exploring Determinants in a Competitive Environment [Article]. *International Journal of Public Administration*, *41*(3), 181- 189. https://doi.org/10.1080/01900692.2016.1256892 |
|  | Guillon, M., Audibert, M., & Mathonnat, J. (2019). La mesure de l'efficience des centres de sante en zone rurale et ses implications: Enseignements tires de la Mongolie. (Assessing the Efficiency of Health Centers in Rural Areas and Its Implications: Lessons from Mongolia. With English summary.). *Revue d'Economie du Developpement*(3), 33-66. |
|  | Guillon, M., Audibert, M., & Mathonnat, J. (2021). Efficiency of district hospitals in Zimbabwe: Assessment, drivers and policy implications [Article in Press]. *The International journal of health planning and management*. https://doi.org/10.1002/hpm.3337 |
|  | Guo, H., Zhao, Y., Niu, T., & Tsui, K.-L. (2017). Hong Kong Hospital Authority resource efficiency evaluation: Via a novel DEA-Malmquist model and Tobit regression model. *PloS one*, *12*(9), e0184211. https://doi.org/10.1371/journal.pone.0184211 |
|  | Hajialiafzali, H., Moss, J. R., & Mahmood, M. A. (2007). Efficiency measurement for hospitals owned by the Iranian social security organisation. *Journal of medical systems*, *31*(3), 166-172. https://doi.org/10.1007/s10916-007-9051-6 |
|  | Halkos, G. E., & Tzeremes, N. G. (2011). A conditional nonparametric analysis for measuring the efficiency of regional public healthcare delivery: an application to Greek prefectures. *Health policy (Amsterdam, Netherlands)*, *103*(1), 73-82. https://doi.org/10.1016/j.healthpol.2010.10.021 |
|  | Halsteinli, V., Kittelsen, S. A., & Magnussen, J. (2010). Productivity growth in outpatient child and adolescent mental health services: the impact of case-mix adjustment. *Social science & medicine (1982)*, *70*(3), 439-446. https://doi.org/10.1016/j.socscimed.2009.11.002 |
|  | Hamidi, S. (2016). Measuring efficiency of governmental hospitals in Palestine using stochastic frontier analysis. *Cost effectiveness and resource allocation : C/E*, *14*, 3. https://doi.org/10.1186/s12962-016-0052-5 |
|  | Hamidi, S., & Akinci, F. (2016). Measuring Efficiency of Health Systems of the Middle East and North Africa (MENA) Region Using Stochastic Frontier Analysis. *Applied health economics and health policy*, *14*(3), 337-347.  https://doi.org/10.1007/s40258-016-0230-9 |
|  | Hamzah, N. M., & See, K. F. (2019). Technical efficiency and its influencing factors in Malaysian hospital pharmacy services. *Health care management science*, *22*(3), 462- 474. https://doi.org/10.1007/s10729-019-09470-8 |
|  | Harrison, J. P., Coppola, M. N., & Wakefield, M. (2004). Efficiency of federal hospitals in the United States. *Journal of medical systems*, *28*(5), 411-422. https://doi.org/10.1023/b:joms.0000041168.28200.8c |
|  | Harrison, J. P., & Lambiase, L. R. (2007). THE IMPROVING EFFICIENCY OF UNIVERSITY HEALTH CONSORTIUM HOSPITALS [Article]. *Journal of Public Budgeting, Accounting & Financial Management*, *19*(3), 385-399.  https://doi.org/10.1108/JPBAFM-19-03-2007-B007 |
|  | Harrison, J. P., Ogniewski, R., & Hoelscher, S. (2009). The improving efficiency of critical access hospitals. *The health care manager*, *28*(3), 209-217. https://doi.org/10.1097/HCM.0b013e3181b3e968 |
|  | Harrison, J. P., & Sexton, C. (2006). The improving efficiency frontier of religious not-for- profit hospitals. *Hospital topics*, *84*(1), 2-10. https://doi.org/10.3200/HTPS.84.1.2-10 |
|  | Harrison, J. P., Spaulding, A., & Mouhalis, P. (2015). The efficiency frontier of for-profit hospitals [Article]. *Journal of health care finance*, *41*(4). |
|  | Hatam, N., Moslehi, S., Askarian, M., Shokrpour, N., Keshtkaran, A., & Abbasi, M. (2010). The efficiency of general public hospitals in Fars Province, Southern Iran [Article]. *Iranian Red Crescent medical journal*, *12*(2), 138-144. |
|  | Hatam, N., Pourmohammadi, K., Keshtkaran, A., Javanbakht, M., & Askarian, M. (2012). Factors affecting efficiency of social security hospitals in Iran: Data envelopment analysis [Article]. *HealthMED*, *6*(6), 1961-1968. |
|  | Helmig, B., & Lapsley, I. (2001). On the efficiency of public, welfare and private hospitals in Germany over time: a sectoral data envelopment analysis study. *Health services management research*, *14*(4), 263-274. https://doi.org/10.1177/095148480101400406 |
|  | Hernández, A. R., & San Sebastián, M. (2014). Assessing the technical efficiency of health posts in rural Guatemala: a data envelopment analysis. *Global health action*, *7*, 23190. https://doi.org/10.3402/gha.v7.23190 |
|  | Herr, A. (2008). Cost and technical efficiency of German hospitals: does ownership matter? *Health economics*, *17*(9), 1057-1071. https://doi.org/10.1002/hec.1388 |
|  | Hofmarcher, M. M., Lietz, C., & Schnabl, A. (2005). Inefficiency in Austrian Inpatient Care: Identifying Ailing Providers Based on DEA Results [Article]. *Central European Journal of Operations Research*, *13*(4), 341-363. |
|  | Hofmarcher, M. M., Paterson, I., & Riedel, M. (2002). Measuring hospital efficiency in Austria--a DEA approach. *Health care management science*, *5*(1), 7-14. https://doi.org/10.1023/a:1013292801100 |
|  | Hollingsworth, B., & Parkin, D. (2001). The efficiency of the delivery of neonatal care in the UK. *Journal of public health medicine*, *23*(1), 47-50. https://doi.org/10.1093/pubmed/23.1.47 |
|  | Hsiao, B., Chen, L.-H., & Wu, H.-T. (2019). Assessing performance of Taiwan hospitals using data envelopment analysis: In view of ownership. *The International journal of health planning and management*, *34*(1), e602-e616. https://doi.org/10.1002/hpm.2676 |
|  | Hsieh, H.-M., Clement, D. G., & Bazzoli, G. J. (2010). Impacts of market and organizational characteristics on hospital efficiency and uncompensated care. *Health care management review*, *35*(1), 77-87. https://doi.org/10.1097/HMR.0b013e3181c09956 |
|  | Hu, H. H., Qi, Q., & Yang, C. H. (2012). Evaluation of China's regional hospital efficiency: DEA approach with undesirable output [Article]. *Journal of the Operational Research Society*, *63*(6), 715-725. https://doi.org/10.1057/jors.2011.77 |
|  | İlgün, G., & Konca, M. (2019). Assessment of efficiency levels of training and research hospitals in Turkey and the factors affecting their efficiencies [Article]. *Health Policy and Technology*, *8*(4), 343-348. https://doi.org/10.1016/j.hlpt.2019.08.008 |
|  | İlgün, G., & Şahin, B. (2020). Investigation of factors affecting efficiency of primary healthcare in Turkey with two-stage data envelopment analysis [Article]. *International Journal of Healthcare Management*, 1-7. https://doi.org/10.1080/20479700.2020.1836735 |
|  | İlgün, G., Sönmez, S., Konca, M., & Yetim, B. (2021). Measuring the efficiency of Turkish maternal and child health hospitals: A two-stage data envelopment analysis. *Evaluation and program planning*, 102023. https://doi.org/10.1016/j.evalprogplan.2021.102023 |
|  | Irwandy, & Sjaaf, A. C. (2018). Using data envelopment analysis to improve the hospitals efficiency in Indonesia: The case of South Sulawesi Province [Article]. *Indian Journal of Public Health Research and Development*, *9*(9), 214-219. https://doi.org/10.5958/0976-5506.2018.00997.X |
|  | Iyengar, R. N., & Ozcan, Y. A. (2009). Performance evaluation of ambulatory surgery centres: an efficiency approach. *Health services management research*, *22*(4), 184- 190. https://doi.org/10.1258/hsmr.2009.009008 |
|  | Jarjue, G., Nor, N. M., Ghani, J. A., & Jalil, S. H. A. (2015). Technical Efficiency of Secondary Health Care Service Delivery in the Gambia [Article]. *International Journal of Economics & Management*, *9*(1), 25-43. |
|  | Jat, T. R., & Sebastian, M. S. (2013). Technical efficiency of public district hospitals in Madhya Pradesh, India: a data envelopment analysis. *Global health action*, *6*, 21742. https://doi.org/10.3402/gha.v6i0.21742 |
|  | Jehu-Appiah, C., Sekidde, S., Adjuik, M., Akazili, J., Almeida, S. D., Nyonator, F., Baltussen, R., Asbu, E. Z., & Kirigia, J. M. (2014). Ownership and technical efficiency of hospitals: evidence from Ghana using data envelopment analysis. *Cost effectiveness and resource allocation : C/E*, *12*(1), 9. https://doi.org/10.1186/1478-7547-12-9 |
|  | Jeremic, V., Bulajic, M., Martic, M., Markovic, A., Savic, G., Jeremic, D., & Radojicic, Z. (2012). An Evaluation of European Countries' Health Systems through Distance Based Analysis. *Hippokratia*, *16*(2), 170-174. |
|  | Jian, W., Huang, Y., Hu, M., & Zhang, X. (2009). Performance evaluation of inpatient service in Beijing: a horizontal comparison with risk adjustment based on Diagnosis Related Groups. *BMC health services research*, *9*, 72.  https://doi.org/10.1186/1472-6963-9-72 |
|  | Jiménez, R. E., Lam, R. M., Marot, M., & Delgado, A. (2004). Observed-predicted length of stay for an acute psychiatric department, as an indicator of inpatient care inefficiencies. Retrospective case-series study. *BMC health services research*, *4*(1), 4. https://doi.org/10.1186/1472-6963-4-4 |
|  | Jing, R., Xu, T., Lai, X., Mahmoudi, E., & Fang, H. (2019). Technical Efficiency of Public and Private Hospitals in Beijing, China: A Comparative Study. *International journal of environmental research and public health*, *17*(1). https://doi.org/10.3390/ijerph17010082 |
|  | Junoy, J. P. (1997). Measuring technical efficiency of output quality in intensive care units. *International journal of health care quality assurance incorporating Leadership in health services*, *10*(2-3), 117-124. https://doi.org/10.1108/09526869710167030 |
|  | Juras, P. E., & Brooks, C. A. (1993). Supporting operational decision making. *The Health care supervisor*, *12*(2), 25-31. |
|  | Kakemam, E., & Dargahi, H. (2019). The Health Sector Evolution Plan and the Technical Efficiency of Public Hospitals in Iran. *Iranian journal of public health*, *48*(9), 1681- 1689. |
|  | Kakeman, E., Rahimi Forushani, A., & Dargahi, H. (2016). Technical Efficiency of Hospitals in Tehran, Iran. *Iranian journal of public health*, *45*(4), 494-502. |
|  | Kalhor, R., Amini, S., Sokhanvar, M., Lotfi, F., Sharifi, M., & Kakemam, E. (2016). Factors affecting the technical efficiency of general hospitals in Iran: data envelopment analysis. *The Journal of the Egyptian Public Health Association*, *91*(1), 20-25. https://doi.org/10.1097/01.EPX.0000480717.13696.3c |
|  | Kalhor, R., Salehi, A., Keshavarz, A., Bastani, P., & Orojloo, P. H. (2014). Assessing Hospital Performance in Iran Using the Pabon Lasso Model [Article]. *Asia Pacific Journal of Health Management*, *9*(2), 77-82. |
|  | Kang, H., Nembhard, H., DeFlitch, C., & Pasupathy, K. (2017). Assessment of emergency department efficiency using data envelopment analysis [Article]. *IISE Transactions on Healthcare Systems Engineering*, *7*(4), 236-246. https://doi.org/10.1080/24725579.2017.1367978 |
|  | Katharaki, M. (2008). Approaching the management of hospital units with an operation research technique: the case of 32 Greek obstetric and gynaecology public units. *Health policy (Amsterdam, Netherlands)*, *85*(1), 19-31. https://doi.org/10.1016/j.healthpol.2007.06.001 |
|  | Kaya Samut, P., & Cafrı, R. (2016). Analysis of the Efficiency Determinants of Health Systems in OECD Countries by DEA and Panel Tobit [Article]. *Social indicators research*, *129*(1), 113-132. https://doi.org/10.1007/s11205-015-1094-3 |
|  | Kazemi, M., Nazari, S., Motamed, N., Arsang-Jang, S., & Fallah, R. (2021). Prediction of Hospitalization Length. Quantile Regression Predicts Hospitalization Length and its Related Factors better than Available Methods. *Annali di igiene : medicina preventiva e di comunita*, *33*(2), 177-188. https://doi.org/10.7416/ai.2021.2423 |
|  | Ketabi, S. (2011). Efficiency measurement of cardiac care units of isfahan hospitals in iran. *Journal of medical systems*, *35*(2), 143-150.  https://doi.org/10.1007/s10916-009-9351-0 |
|  | Khushalani, J., & Ozcan, Y. A. (2017). Are hospitals producing quality care efficiently? An analysis using Dynamic Network Data Envelopment Analysis (DEA) [Article]. *Socio- economic planning sciences*, *60*, 15-23. https://doi.org/10.1016/j.seps.2017.01.009 |
|  | Kim, Y., Lee, K.-H., & Choi, S. W. (2021). Multifaced Evidence of Hospital Performance in Pennsylvania. *Healthcare (Basel, Switzerland)*, *9*(6). https://doi.org/10.3390/healthcare9060670 |
|  | Kinfu, Y. (2013). The efficiency of the health system in South Africa: evidence from stochastic frontier analysis [Article]. *Applied Economics*, *45*(8), 1003-1010. https://doi.org/10.1080/00036846.2011.613787 |
|  | Kirigia, J. M., & Asbu, E. Z. (2013). Technical and scale efficiency of public community hospitals in Eritrea: an exploratory study. *Health economics review*, *3*(1), 6. https://doi.org/10.1186/2191-1991-3-6 |
|  | Kirigia, J. M., Emrouznejad, A., & Sambo, L. G. (2002). Measurement of technical efficiency of public hospitals in Kenya: using Data Envelopment Analysis. *Journal of medical systems*, *26*(1), 39-45. https://doi.org/10.1023/a:1013090804067 |
|  | Kirigia, J. M., Emrouznejad, A., Sambo, L. G., Munguti, N., & Liambila, W. (2004). Using data envelopment analysis to measure the technical efficiency of public health centers in Kenya. *Journal of medical systems*, *28*(2), 155-166. https://doi.org/10.1023/b:joms.0000023298.31972.c9 |
|  | Kirigia, J. M., Sambo, L. G., & Scheel, H. (2001). Technical efficiency of public clinics in Kwazulu-Natal Province of South Africa. *East African medical journal*, *78*(3 Suppl), S1-S13. https://doi.org/10.4314/eamj.v78i3.9070 |
|  | Kjekshus, L. E., & Hagen, T. P. (2005). Ring fencing of elective surgery: does it affect hospital efficiency? *Health services management research*, *18*(3), 186-197. https://doi.org/10.1258/0951484054572529 |
|  | Kjellstrand, C. M., Kovithavongs, C., & Szabo, E. (1998). On the success, cost and efficiency of modern medicine: an international comparison. *Journal of internal medicine*, *243*(1), 3-14. https://doi.org/10.1046/j.1365-2796.1998.00248.x |
|  | Konstante, R. (2013). THE APPLICATION OF THE DATA ENVELOPMENT ANALYSIS METHOD TO EVALUATION AND PLANNING OF HOSPITAL EFFICIENCY IN LATVIA [Article]. *Journal of Economics & Management Research*, *2*, 79-94. |
|  | Kontodimopoulos, N., Bellali, T., Labiris, G., & Niakas, D. (2006). Investigating sources of inefficiency in residential mental health facilities [Article]. *Journal of medical systems*, *30*(3), 169-176. https://doi.org/10.1007/s10916-005-7981-4 |
|  | Kontodimopoulos, N., Moschovakis, G., Aletras, V. H., & Niakas, D. (2007). The effect of environmental factors on technical and scale efficiency of primary health care providers in Greece. *Cost effectiveness and resource allocation : C/E*, *5*, 14. https://doi.org/10.1186/1478-7547-5-14 |
|  | Kourtis, M., Curtis, P., Hanias, M., & Kourtis, E. (2021). A Strategic Financial Management Evaluation of Private Hospitals' Effectiveness and Efficiency for Sustainable Financing: A Research Study. *European Research Studies*, *24*(1), 1025-1054. https://doi.org/https://www.ersj.eu/index.php |
|  | Kreng, V. B., Yang, S.-w., & Lin, C.-H. (2014). Measuring health care efficiency with a tripartite configuration under the "National" Health Insurance system. *Chinese medical journal*, *127*(9), 1633-1639. |
|  | Kristensen, T., Bogetoft, P., & Pedersen, K. M. (2010). Potential gains from hospital mergers in Denmark. *Health care management science*, *13*(4), 334-345. https://doi.org/10.1007/s10729-010-9133-8 |
|  | Küçük, A., Özsoy, V. S., & Balkan, D. (2020). Assessment of technical efficiency of public hospitals in Turkey. *European journal of public health*, *30*(2), 230-235. https://doi.org/10.1093/eurpub/ckz143 |
|  | Kumar, S., & Nunne, W. H. (2008). Measuring technical efficiency of specialty hospitals in the US [Article]. *Journal of Revenue & Pricing Management*, *7*(2), 139-152. https://doi.org/10.1057/rpm.2008.6 |
|  | Kuwabara, K., Matsuda, S., Fushimi, K., Ishikawa, K. B., Horiguchi, H., Hayashida, K., & Fujimori, K. (2011). Contribution of case-mix classification to profiling hospital characteristics and productivity. *The International journal of health planning and management*, *26*(3), e138-e150. https://doi.org/10.1002/hpm.1051 |
|  | Laine, J., Finne-Soveri, U. H., Björkgren, M., Linna, M., Noro, A., & Häkkinen, U. (2005). The association between quality of care and technical efficiency in long-term care. *International journal for quality in health care : journal of the International Society for Quality in Health Care*, *17*(3), 259-267. https://doi.org/10.1093/intqhc/mzi032 |
|  | Laine, J., Linna, M., Häkkinen, U., & Noro, A. (2005). Measuring the productive efficiency and clinical quality of institutional long-term care for the elderly. *Health economics*, *14*(3), 245-256. https://doi.org/10.1002/hec.926 |
|  | Lari, M. S., & Sefiddashti, S. E. (2021). Measuring the Efficiency of Health Systems: A Case of Mental Health in Middle East and North Africa Countries. *Iranian journal of public health*, *50*(5), 1017-1027. https://doi.org/10.18502/ijph.v50i5.6119 |
|  | Lee, K., & Wan, T. T. H. (2002). Effects of hospitals' structural clinical integration on efficiency and patient outcome. *Health services management research*, *15*(4), 234- 244. https://doi.org/10.1258/095148402320589037 |
|  | Lee, K.-H., Park, J., Lim, S., & Park, S.-C. (2015). Has competition increased hospital technical efficiency? *The health care manager*, *34*(2), 106-112. https://doi.org/10.1097/HCM.0000000000000061 |
|  | Lee, K.-H., Yang, S.-B., & Choi, M. (2009). The association between hospital ownership and technical efficiency in a managed care environment. *Journal of medical systems*, *33*(4), 307-315. https://doi.org/10.1007/s10916-008-9192-2 |
|  | Lee, K.-s., Chun, K.-H., & Lee, J.-S. (2008). Reforming the hospital service structure to improve efficiency: urban hospital specialization. *Health policy (Amsterdam, Netherlands)*, *87*(1), 41-49. https://doi.org/10.1016/j.healthpol.2007.10.003 |
|  | Lee, Y. J., & Joo, S.-J. (2020). Assessing the effects of exogenous factors for benchmarking hospitals with double bootstrapping [Article]. *Benchmarking: An International Journal*, *27*(1), 250-263. https://doi.org/10.1108/BIJ-01-2018-0005 |
|  | Leleu, H., Al-Amin, M., Rosko, M., & Valdmanis, V. G. (2018). A robust analysis of hospital efficiency and factors affecting variability. *Health services management research*, *31*(1), 33-42. https://doi.org/10.1177/0951484817730537 |
|  | Leu, R. (1978). Ansatze zur empirischen Messung der relativen Effizienz von Gesundheitssystemen. (Empirically Measuring the Relative Efficiency of Health Care Systems. With English summary.). *Schweizerische Zeitschrift fur Volkswirtschaft und Statistik*, *114*(3), 479-503. |
|  | Leu, R. (1978). [Economic aspects of prevention. Efficiency and structure of the health care system]. *Sozial- und Praventivmedizin*, *23*(5-6), 335-340. https://doi.org/10.1007/BF02074219 |
|  | Li, B., Mohiuddin, M., & Liu, Q. (2019). Determinants and Differences of Township Hospital Efficiency among Chinese Provinces. *International journal of environmental research and public health*, *16*(9). https://doi.org/10.3390/ijerph16091601 |
|  | Li, L., & Liu, Z. (2021). Research on efficiency measurement and spatiotemporal disparity of rural public health services in China. *PloS one*, *16*(7), e0252871. https://doi.org/10.1371/journal.pone.0252871 |
|  | Li, N.-N., Wang, C.-H., Ni, H., & Wang, H. (2017). Efficiency and Productivity of County- level Public Hospitals Based on the Data Envelopment Analysis Model and Malmquist Index in Anhui, China. *Chinese medical journal*, *130*(23), 2836-2843. https://doi.org/10.4103/0366-6999.219148 |
|  | Li, Q., Tian, L., Jing, X., Chen, X., Li, J., & Chen, H. (2020). Efficiency and scale effect of county public hospitals in Shandong Province, China: a cross-sectional study. *BMJ open*, *10*(6), e035703. https://doi.org/10.1136/bmjopen-2019-035703 |
|  | Li, Z., Yang, L., Tang, S., & Bian, Y. (2020). Equity and Efficiency of Health Resource Allocation of Chinese Medicine in Mainland China: 2013-2017. *Frontiers in public health*, *8*, 579269. https://doi.org/10.3389/fpubh.2020.579269 |
|  | Li, Z., Zhang, W., Kong, A., Ding, Z., Wei, H., & Guo, Y. (2021). Configuration Analysis of Influencing Factors of Technical Efficiency Based on DEA and fsQCA: Evidence from China's Medical and Health Institutions. *Risk management and healthcare policy*, *14*, 49-65. https://doi.org/10.2147/RMHP.S282178 |
|  | Lin, L., Wu, F., Chen, W., Zhu, C., & Huang, T. (2021). Research on Urban Medical and Health Services Efficiency and Its Spatial Correlation in China: Based on Panel Data of 13 Cities in Jiangsu Province. *Healthcare (Basel, Switzerland)*, *9*(9). https://doi.org/10.3390/healthcare9091167 |
|  | Lindlbauer, I., & Schreyögg, J. (2014). The relationship between hospital specialization and hospital efficiency: do different measures of specialization lead to different results? *Health care management science*, *17*(4), 365-378. https://doi.org/10.1007/s10729-014-9275-1 |
|  | Linna, M. (1998). Measuring hospital cost efficiency with panel data models. *Health economics*, *7*(5), 415-427.  https://doi.org/10.1002/(sici)1099-1050(199808)7:5<415::aid-hec357>3.0.co;2-9 |
|  | Linna, M., Häkkinen, U., Peltola, M., Magnussen, J., Anthun, K. S., Kittelsen, S., Roed, A., Olsen, K., Medin, E., & Rehnberg, C. (2010). Measuring cost efficiency in the Nordic hospitals--a cross-sectional comparison of public hospitals in 2002. *Health care management science*, *13*(4), 346-357. https://doi.org/10.1007/s10729-010-9134-7 |
|  | Lobo, M. S. C., Ozcan, Y. A., Lins, M. P. E., Silva, A. C. M., & Fiszman, R. (2014). Teaching hospitals in Brazil: Findings on determinants for efficiency [Article]. *International Journal of Healthcare Management*, *7*(1), 60-68. https://doi.org/10.1179/2047971913Y.0000000055 |
|  | Longo, F., Siciliani, L., Gravelle, H., & Santos, R. (2017). Do hospitals respond to rivals' quality and efficiency? A spatial panel econometric analysis. *Health economics*, *26 Suppl 2*, 38-62. https://doi.org/10.1002/hec.3569 |
|  | Lotfi, F., Kalhor, R., Bastani, P., Shaarbafchi Zadeh, N., Eslamian, M., Dehghani, M. R., & Kiaee, M. Z. (2014). Various indicators for the assessment of hospitals' performance status: differences and similarities. *Iranian Red Crescent medical journal*, *16*(4), e12950. https://doi.org/10.5812/ircmj.12950 |
|  | Lu, W., Evans, R. D., Zhang, T., Ni, Z., & Tao, H. (2020). Evaluation of resource utilization efficiency in obstetrics and gynecology units in China: A three-stage data envelopment analysis of the Shanxi province. *The International journal of health planning and management*, *35*(1), 309-317. https://doi.org/10.1002/hpm.2908 |
|  | Luoma, K., Järviö, M. L., Suoniemi, I., & Hjerppe, R. T. (1996). Financial incentives and productive efficiency in Finnish health centres. *Health economics*, *5*(5), 435-445. https://doi.org/10.1002/(SICI)1099-1050(199609)5:5<435::AID-HEC223>3.0.CO;2- Y |
|  | Lynch, J. R., & Ozcan, Y. A. (1994). Hospital closure: an efficiency analysis. *Hospital & health services administration*, *39*(2), 205-220. |
|  | Lyroudi, K., Glaveli, N., Koulakiotis, A., & Angelidis, D. (2006). The productive performance of public hospital clinics in Greece: a case study. *Health services management research*, *19*(2), 67-72. https://doi.org/10.1258/095148406776829059 |
|  | Magnussen, J., & Nyland, K. (2008). Measuring efficiency in clinical departments. *Health policy (Amsterdam, Netherlands)*, *87*(1), 1-7. https://doi.org/10.1016/j.healthpol.2007.09.013 |
|  | Manavgat, G., & DemİRcİ, A. (2020). Decentralization Matter of Healthcare and Effect on Regional Healthcare Efficiency: Evidence from Turkey [Article]. *Sağlık Hizmetlerinde Yerinden Yönetimin Hizmet Performansı Üzerindeki Etkisi: Türkiye Örneği.*, *28*(44), 261-282. https://doi.org/10.17233/sosyoekonomi.2020.02.12 |
|  | Mancuso, P., & Valdmanis, V. G. (2016). Care Appropriateness and Health Productivity Evolution: A Non-Parametric Analysis of the Italian Regional Health Systems. *Applied health economics and health policy*, *14*(5), 595-607. https://doi.org/10.1007/s40258-016-0257-y |
|  | Maniadakis, N., Kotsopoulos, N., Prezerakos, P., & Yfantopoulos, J. (2008). Measuring Intra-hospital Clinic Efficiency and Productivity: An Application to a Greek University General Hospital. *European Research Studies*, *11*(1-2), 95-109. |
|  | Marathe, S., Wan, T. T. H., Zhang, J., & Sherin, K. (2007). Factors influencing community health centers' efficiency: a latent growth curve modeling approach. *Journal of medical systems*, *31*(5), 365-374. https://doi.org/10.1007/s10916-007-9078-8 |
|  | Marschall, P., & Flessa, S. (2011). Efficiency of primary care in rural Burkina Faso. A two- stage DEA analysis. *Health economics review*, *1*(1), 5.  https://doi.org/10.1186/2191-1991-1-5 |
|  | Martinussen, P. E., & Midttun, L. (2004). Day surgery and hospital efficiency: empirical analysis of Norwegian hospitals, 1999-2001. *Health policy (Amsterdam, Netherlands)*, *68*(2), 183-196. https://doi.org/10.1016/j.healthpol.2003.09.003 |
|  | Masiye, F. (2007). Investigating health system performance: an application of data envelopment analysis to Zambian hospitals. *BMC health services research*, *7*, 58. https://doi.org/10.1186/1472-6963-7-58 |
|  | Mateus, C., Joaquim, I., & Nunes, C. (2015). Measuring hospital efficiency--comparing four European countries. *European journal of public health*, *25 Suppl 1*, 52-58. https://doi.org/10.1093/eurpub/cku222 |
|  | Matos, R., Ferreira, D., & Pedro, M. I. (2021). Economic Analysis of Portuguese Public Hospitals Through the Construction of Quality, Efficiency, Access, and Financial Related Composite Indicators. *Social indicators research*, 1-32. https://doi.org/10.1007/s11205-021-02650-6 |
|  | McGarvey, R. G., Thorsen, A., Thorsen, M. L., & Reddy, R. M. (2019). Measuring efficiency of community health centers: a multi-model approach considering quality of care and heterogeneous operating environments [Article]. *Health care management science*, *22*(3), 489-511. https://doi.org/10.1007/s10729-018-9455-5 |
|  | McGuire, A. (1987). The measurement of hospital efficiency. *Social science & medicine (1982)*, *24*(9), 719-724. https://doi.org/10.1016/0277-9536(87)90108-0 |
|  | McKillop, D. G. (1999). Efficiency in Northern Ireland Hospitals: A Non-parametric Analysis. *Economic and Social Review*, *30*(2), 175-196. https://doi.org/http://www.esr.ie/issue/archive |
|  | Medin, E., Anthun, K. S., Häkkinen, U., Kittelsen, S. A. C., Linna, M., Magnussen, J., Olsen, K., & Rehnberg, C. (2011). Cost efficiency of university hospitals in the Nordic countries: a cross-country analysis. *The European journal of health economics : HEPAC : health economics in prevention and care*, *12*(6), 509-519.  https://doi.org/10.1007/s10198-010-0263-1 |
|  | Mennicken, R., Kuntz, L., & Schwierz, C. (2011). The trade-off between efficiency and quality in hospital departments. *Journal of health organization and management*, *25*(5), 564-577. https://doi.org/10.1108/14777261111161897 |
|  | Michel, M., Alberti, C., Carel, J. C., & Chevreul, K. (2019). Association of Pediatric Inpatient Socioeconomic Status with Hospital Efficiency and Financial Balance [Article in Press]. *JAMA Network Open*. https://doi.org/10.1001/jamanetworkopen.2019.13656 |
|  | Michel, M., Alberti, C., Carel, J.-C., & Chevreul, K. (2020). Socioeconomic Status of Newborns and Hospital Efficiency: Implications for Hospital Payment Methods. *Value in health : the journal of the International Society for Pharmacoeconomics and Outcomes Research*, *23*(3), 335-342. https://doi.org/10.1016/j.jval.2019.10.008 |
|  | Miclos, P. V., Calvo, M. C. M., & Colussi, C. F. (2017). Evaluation of the performance of actions and outcomes in primary health care. *Revista de saude publica*, *51*, 86. https://doi.org/10.11606/S1518-8787.2017051006831 |
|  | Milliken, O., Devlin, R. A., Barham, V., Hogg, W., Dahrouge, S., & Russell, G. (2011). Comparative efficiency assessment of primary care service delivery models using data envelopment analysis. *Canadian public policy. Analyse de politiques*, *37*(1), 85- 109. https://doi.org/10.3138/cpp.37.1.85 |
|  | Mitropoulos, P., Kounetas, K., & Mitropoulos, I. (2016). Factors affecting primary health care centers' economic and production efficiency [Article]. *Annals of Operations Research*, *247*(2), 807-822. https://doi.org/10.1007/s10479-015-2056-5 |
|  | Mitropoulos, P., Mitropoulos, I., & Sissouras, A. (2013). Managing for efficiency in health care: the case of Greek public hospitals. *The European journal of health economics : HEPAC : health economics in prevention and care*, *14*(6), 929-938. https://doi.org/10.1007/s10198-012-0437-0 |
|  | Mitrović, Z., Vujošević, M., & Savić, G. (2015). Data Envelopment Analysis for Evaluating Serbia's Health Care System [Article]. *Management (1820-0222)*, *20*(75), 39-46. https://doi.org/10.7595/management.fon.2015.0012 |
|  | Mobley, L. R., & Magnussen, J. (1998). An international comparison of hospital efficiency: does institutional environment matter? [Article]. *Applied Economics*, *30*(8), 1089- 1100. https://doi.org/10.1080/000368498325255 |
|  | Mogha, S. K., Yadav, S. P., & Singh, S. P. (2015). Slack based measure of efficiencies of public sector hospitals in Uttarakhand (India) [Article]. *Benchmarking: An International Journal*, *22*(7), 1229-1246. https://doi.org/10.1108/BIJ-12-2013-0122 |
|  | Mohamadi, E., Olyaee Manesh, A., Takian, A., Majdzadeh, R., Hosseinzadeh Lotfi, F., Sharafi, H., Jowett, M., Kiani, M. M., Hosseini Qavam Abadi, L., Fazaeli, A. A., Goodarzi, Z., Sajadi, H. S., Noori Hekmat, S., & Freidoony, L. (2020). Technical efficiency in health production: A comparison between Iran and other upper middle- income countries [Article]. *Health Policy and Technology*, *9*(3), 335-347. https://doi.org/10.1016/j.hlpt.2020.06.007 |
|  | Mohammadpour, S., Javan-Noughabi, J., Vafaee Najar, A., Zangeneh, M., Yousefi, S., Nouhi, M., & Jahangiri, R. (2020). Factors affecting the technical efficiency of rural primary health care centers in Hamadan, Iran: data envelopment analysis and Tobit regression. *Cost effectiveness and resource allocation : C/E*, *18*(1), 53. https://doi.org/10.1186/s12962-020-00249-1 |
|  | Moran, V., & Jacobs, R. (2013). An international comparison of efficiency of inpatient mental health care systems. *Health policy (Amsterdam, Netherlands)*, *112*(1-2), 88- 99. https://doi.org/10.1016/j.healthpol.2013.06.011 |
|  | Morey, R. C., & Dittman, D. A. (1996). Cost pass-through reimbursement to hospitals and their impacts on operating efficiencies [Article]. *Annals of Operations Research*, *67*(1-4), 117-139. https://doi.org/10.1007/BF02187026 |
|  | Morse, E. V., Gordon, G., & Moch, M. (1974). Hospital costs and quality of care. An organizational perspective [Article]. *MILBANK MEM.FD QUART.*, *52*(3), 315-346. https://doi.org/10.2307/3349639 |
|  | Mousa, W., & Aldehayyat, J. S. (2018). Regional efficiency of healthcare services in Saudi Arabia [Article]. *Middle East Development Journal*, *10*(1), 152-174. https://doi.org/10.1080/17938120.2018.1443607 |
|  | Mujasi, P. N., Asbu, E. Z., & Puig-Junoy, J. (2016). How efficient are referral hospitals in Uganda? A data envelopment analysis and tobit regression approach. *BMC health services research*, *16*, 230. https://doi.org/10.1186/s12913-016-1472-9 |
|  | Navarro-Espigares, J., & Torres, E. (2011). Efficiency and quality in health services: a crucial link [Article]. *Service Industries Journal*, *31*(3), 385-403. https://doi.org/10.1080/02642060802712798 |
|  | Nayar, P., & Ozcan, Y. A. (2008). Data envelopment analysis comparison of hospital efficiency and quality. *Journal of medical systems*, *32*(3), 193-199. https://doi.org/10.1007/s10916-007-9122-8 |
|  | Nayar, P., Ozcan, Y. A., Yu, F., & Nguyen, A. T. (2013). Benchmarking urban acute care hospitals: efficiency and quality perspectives. *Health care management review*, *38*(2), 137-145. https://doi.org/10.1097/HMR.0b013e3182527a4c |
|  | Nedelea, I. C., & Fannin, J. M. (2017). Testing for cost efficiency differences between two groups of rural hospitals [Article]. *International Journal of Healthcare Management*, *10*(1), 57-65. https://doi.org/10.1080/20479700.2016.1259146 |
|  | Ng, Y. C. (2008). The Productive Efficiency of the Health Care Sector of China. *Review of Regional Studies*, *38*(3), 381-393. |
|  | Ngobeni, V., Breitenbach, M. C., & Aye, G. C. (2020). Technical efficiency of provincial public healthcare in South Africa. *Cost effectiveness and resource allocation : C/E*, *18*, 3. https://doi.org/10.1186/s12962-020-0199-y |
|  | Ni Luasa, S., Dineen, D., & Zieba, M. (2018). Technical and scale efficiency in public and private Irish nursing homes - a bootstrap DEA approach. *Health care management science*, *21*(3), 326-347. https://doi.org/10.1007/s10729-016-9389-8 |
|  | Novignon, J., & Nonvignon, J. (2017). Improving primary health care facility performance in Ghana: efficiency analysis and fiscal space implications. *BMC health services research*, *17*(1), 399. https://doi.org/10.1186/s12913-017-2347-4 |
|  | Nunamaker, T. R. (1983). Measuring routine nursing service efficiency: a comparison of cost per patient day and data envelopment analysis models. *Health services research*, *18*(2 Pt 1), 183-208. |
|  | Nundoochan, A. (2020). Improving public hospital efficiency and fiscal space implications: the case of Mauritius. *International journal for equity in health*, *19*(1), 152. https://doi.org/10.1186/s12939-020-01262-9 |
|  | Nuti, S., Daraio, C., Speroni, C., & Vainieri, M. (2011). Relationships between technical efficiency and the quality and costs of health care in Italy. *International journal for quality in health care : journal of the International Society for Quality in Health Care*, *23*(3), 324-330. https://doi.org/10.1093/intqhc/mzr005 |
|  | Oikonomou, N., Tountas, Y., Mariolis, A., Souliotis, K., Athanasakis, K., & Kyriopoulos, J. (2016). Measuring the efficiency of the Greek rural primary health care using a restricted DEA model; the case of southern and western Greece. *Health care management science*, *19*(4), 313-325. https://doi.org/10.1007/s10729-015-9324-4 |
|  | Olsen, K. R., Gyrd-Hansen, D., Sørensen, T. H., Kristensen, T., Vedsted, P., & Street, A. (2013). Organisational determinants of production and efficiency in general practice: a population-based study. *The European journal of health economics : HEPAC : health economics in prevention and care*, *14*(2), 267-276.  https://doi.org/10.1007/s10198-011-0368-1 |
|  | Onady, G. M. (1997). A community collaborative practice experience between Med/Peds and family practice. *The American journal of medicine*, *102*(5), 441-448. https://doi.org/10.1016/S0002-9343(97)00005-3 |
|  | Onder, O., Cook, W., & Kristal, M. (2022). Does quality help the financial viability of hospitals? A data envelopment analysis approach [Article]. *Socio-economic planning sciences*, *79*, N.PAG-N.PAG. https://doi.org/10.1016/j.seps.2021.101105 |
|  | Ortega-Díaz, M. I., Ocaña-Riola, R., Pérez-Romero, C., & Martín-Martín, J. J. (2020). Multilevel Analysis of the Relationship between Ownership Structure and Technical Efficiency Frontier in the Spanish National Health System Hospitals. *International journal of environmental research and public health*, *17*(16). https://doi.org/10.3390/ijerph17165905 |
|  | Ortiz, J., Meemon, N., Tang, C.-Y., Wan, T. T. H., & Paek, S. C. (2011). Rural Health Clinic efficiency and effectiveness: insight from a nationwide survey. *Journal of medical systems*, *35*(4), 671-681. https://doi.org/10.1007/s10916-009-9404-4 |
|  | Ozcan, Y. A., Luke, R. D., & Haksever, C. (1992). Ownership and organizational performance. A comparison of technical efficiency across hospital types. *Medical care*, *30*(9), 781-794. |
|  | Ozcan, Y. A., McCue, M. J., & Okasha, A. A. (1996). Measuring the technical efficiency of psychiatric hospitals. *Journal of medical systems*, *20*(3), 141-150. https://doi.org/10.1007/BF02281992 |
|  | Ozcan, Y. A., Shukla, R. K., & Tyler, L. H. (1997). Organizational Performance in the Community Mental Health Care System: The Need Fulfillment Perspective [Article]. *Organization Science*, *8*(2), 176-191. https://doi.org/10.1287/orsc.8.2.176 |
|  | Ozgen, H., & Sahin, I. (2010). Measurement of efficiency of the dialysis sector in Turkey using data envelopment analysis. *Health policy (Amsterdam, Netherlands)*, *95*(2-3), 185-193. https://doi.org/10.1016/j.healthpol.2009.11.020 |
|  | Paddock, S. M., Damberg, C. L., Yanagihara, D., Adams, J. L., Burgette, L., & Escarce, J. J. (2017). What Role Does Efficiency Play in Understanding the Relationship Between Cost and Quality in Physician Organizations? *Medical care*, *55*(12), 1039-1045. https://doi.org/10.1097/MLR.0000000000000823 |
|  | Pai, D. R., Hosseini, H., & Brown, R. S. (2017). Does efficiency and quality of care affect hospital closures? *Health systems (Basingstoke, England)*, *8*(1), 17-30. https://doi.org/10.1080/20476965.2017.1405874 |
|  | Pantouvakis, A., & Mpogiatzidis, P. (2013). Measuring clinical department efficiency – the impact of clinical leadership job satisfactionAn application to public hospitals [Article]. *Benchmarking: An International Journal*, *20*(3), 305-321. https://doi.org/10.1108/14635771311318108 |
|  | Papadaki, S., & Stankova, P. (2016). Comparison of Horizontally Integrated Hospitals in Private and Public Sectors of Czech Republic. *Economics and Sociology*, *9*(3), 180- 194. https://doi.org/http://www.economics-sociology.eu/ |
|  | Pelone, F., Kringos, D. S., Valerio, L., Romaniello, A., Lazzari, A., Ricciardi, W., & Giulio de Belvis, A. (2012). The measurement of relative efficiency of general practice and the implications for policy makers. *Health policy (Amsterdam, Netherlands)*, *107*(2- 3), 258-268. https://doi.org/10.1016/j.healthpol.2012.05.005 |
|  | Peng, Z., Zhu, L., Wan, G., & Coyte, P. C. (2021). Can integrated care improve the efficiency of hospitals? Research based on 200 Hospitals in China. *Cost effectiveness and resource allocation : C/E*, *19*(1), 61. https://doi.org/10.1186/s12962-021-00314-3 |
|  | Pham, T. L. (2011). Efficiency and productivity of hospitals in Vietnam. *Journal of health organization and management*, *25*(2), 195-213. https://doi.org/10.1108/14777261111134428 |
|  | Pilyavsky, A., & Staat, M. (2008). Efficiency and productivity change in Ukrainian health care [Article]. *Journal of Productivity Analysis*, *29*(2), 143-154. https://doi.org/10.1007/s11123-007-0070-6 |
|  | Pink, G. H., Murray, M. A., & McKillop, I. (2003). Hospital efficiency and patient satisfaction. *Health services management research*, *16*(1), 24-38. https://doi.org/10.1258/095148403762539112 |
|  | Piubello Orsini, L., Leardini, C., Vernizzi, S., & Campedelli, B. (2021). Inefficiency of public hospitals: a multistage data envelopment analysis in an Italian region. *BMC health services research*, *21*(1), 1281. https://doi.org/10.1186/s12913-021-07276-5 |
|  | Polisena, J., Laporte, A., Coyte, P. C., & Croxford, R. (2010). Performance evaluation in home and community care. *Journal of medical systems*, *34*(3), 291-297. https://doi.org/10.1007/s10916-008-9240-y |
|  | Polyzos, N. (2012). A three-year Performance Evaluation of the NHS Hospitals in Greece. *Hippokratia*, *16*(4), 350-355. |
|  | Potter, S. J. (2001). A longitudinal analysis of the distinction between for-profit and not-for- profit hospitals in America. *Journal of health and social behavior*, *42*(1), 17-44. |
|  | Pratt, W. R. (2010). What does free cash flow tell us about hospital efficiency? A stochastic frontier analysis of cost inefficiency in California hospitals. *Journal of health care finance*, *37*(1), 35-44. |
|  | Purohit, B. C. (2010). Efficiency of health care system at the sub-state level in Madhya Pradesh, India. *Social work in public health*, *25*(1), 42-58. https://doi.org/10.1080/19371910902877217 |
|  | Qureshi, W., & Hassan, G. (2014). A five year retrospective study of bed utilization trends in a tertiary care teaching institution [Article]. *JK Science*, *16*(3), 119-121. |
|  | Ramanathan, R. (2005). Operations assessment of hospitals in the Sultanate of Oman [Article]. *International Journal of Operations & Production Management*, *25*(1), 39- 54. https://doi.org/10.1108/01443570510572231 |
|  | Ramanathan, T. V., Chandra, K. S., & Thupeng, W. M. (2003). A comparison of the technical efficiencies of health districts and hospitals in Botswana [Article]. *Development Southern Africa*, *20*(2), 307. https://doi.org/10.1080/03768350302955 |
|  | Ramírez-Valdivia, M., Bustos, J., Maturana, S., & Mendoza-Alonzo, J. (2015). Measuring the Efficiency of Chilean Primary Healthcare Centres [Article]. *International Journal of Engineering Business Management*, *7*, 1-10. https://doi.org/10.5772/60839 |
|  | Rattanachotphanit, T., Limwattananon, C., Limwattananon, S., Johns, J. R., Schommer, J. C., & Brown, L. M. (2008). Assessing the efficiency of hospital pharmacy services in Thai public district hospitals. *The Southeast Asian journal of tropical medicine and public health*, *39*(4), 753-765. |
|  | Ravangard, R., Hatam, N., Teimourizad, A., & Jafari, A. (2014). Factors affecting the technical efficiency of health systems: A case study of Economic Cooperation Organization (ECO) countries (2004-10). *International journal of health policy and management*, *3*(2), 63-69. https://doi.org/10.15171/ijhpm.2014.60 |
|  | Razzaq, S., Ali Chaudhary, A., & Razzaq Khan, A. (2013). Efficiency Analysis of Basic Health Units: A Comparison of Developed and Deprived Regions in Azad Jammu and Kashmir. *Iranian journal of public health*, *42*(11), 1223-1231. |
|  | Register, C. A., & Bruning, E. R. (1987). Profit Incentives and Technical Efficiency in the Production of Hospital Care [Article]. *Southern Economic Journal*, *53*(4), 899. https://doi.org/10.2307/1059684 |
|  | Renner, A., Kirigia, J. M., Zere, E. A., Barry, S. P., Kirigia, D. G., Kamara, C., & Muthuri, L. H. K. (2005). Technical efficiency of peripheral health units in Pujehun district of Sierra Leone: a DEA application. *BMC health services research*, *5*, 77. https://doi.org/10.1186/1472-6963-5-77 |
|  | Rezaee, M. J., & Karimdadi, A. (2015). Do Geographical Locations Affect in Hospitals Performance? A Multi-group Data Envelopment Analysis. *Journal of medical systems*, *39*(9), 85. https://doi.org/10.1007/s10916-015-0278-3 |
|  | Rezaei, S., Zandian, H., Baniasadi, A., Moghadam, T. Z., Delavari, S., & Delavari, S. (2016). Measuring the Efficiency of a Hospital based on the Econometric Stochastic Frontier Analysis (SFA) Method. *Electronic physician*, *8*(2), 2025-2029. https://doi.org/10.19082/2025 |
|  | Rezapour, A., Ebadifard Azar, F., Yousef Zadeh, N., Roumiani, Y., & Bagheri Faradonbeh, S. (2015). Technical efficiency and resources allocation in university hospitals in Tehran, 2009-2012. *Medical journal of the Islamic Republic of Iran*, *29*, 266. |
|  | Rezapour, A., Foroughi, Z., Sadeghi, N. S., Faraji, M., Mazdaki, A., Asiabar, A. S., Niknam, N., Rahmani, K., & Mohammad-Pour, S. (2019). Identification of the most appropriate variables for measuring the efficiency of Iranian public hospitals: Using Delphi technique. *Journal of education and health promotion*, *8*, 140. https://doi.org/10.4103/jehp.jehp_402_18 |
|  | Roh, C.-Y., Moon, M. J., & Jung, K. (2013). Efficiency disparities among community hospitals in Tennessee: do size, location, ownership, and network matter? *Journal of health care for the poor and underserved*, *24*(4), 1816-1833. https://doi.org/10.1353/hpu.2013.0175 |
|  | Rollins, J., Lee, K., Xu, Y., & Ozcan, Y. A. (2001). Longitudinal study of health maintenance organization efficiency. *Health services management research*, *14*(4), 249-262. https://doi.org/10.1177/095148480101400405 |
|  | Rosenman, R., Siddharthan, K., & Ahern, M. (1997). Output efficiency of health maintenance organizations in Florida. *Health economics*, *6*(3), 295-302. https://doi.org/10.1002/(sici)1099-1050(199705)6:3<295::aid-hec265>3.0.co;2-# |
|  | Rosko, M., Al-Amin, M., & Tavakoli, M. (2020). Efficiency and profitability in US not-for- profit hospitals. *International journal of health economics and management*, *20*(4), 359-379. https://doi.org/10.1007/s10754-020-09284-0 |
|  | Rosko, M. D., Chilingerian, J. A., Zinn, J. S., Aaronson, W. E., Rosko, M. D., Chilingerian, J. A., Zinn, J. S., & Aaronson, W. E. (1995). The effects of ownership, operating environment, and strategic choices on nursing home efficiency. *Medical care*, *33*(10), 1001-1021. |
|  | Rosko, M. D., & Mutter, R. L. (2010). Inefficiency differences between critical access hospitals and prospectively paid rural hospitals. *Journal of health politics, policy and law*, *35*(1), 95-126. https://doi.org/10.1215/03616878-2009-042 |
|  | Sabermahani, A., Ghaderi, H., & Barouni, M. (2012). Measuring the technical efficiency of hospitals in Iran: Case of Kerman's province: 2011 [Article]. *HealthMED*, *6*(7), 2569-2576. |
|  | Şahin, B., İlgün, G., & Sönmez, S. (2021). Determining the factors affecting the technical efficiency scores of public hospitals using different regression methods [Article]. *Benchmarking: An International Journal*, *28*(7), 2202-2215. https://doi.org/10.1108/BIJ-08-2020-0427 |
|  | Sandiford, P., Consuelo, D. J. J. V., & Rouse, P. (2017). How efficient are New Zealand's District Health Boards at producing life expectancy gains for Māori and Europeans? *Australian and New Zealand journal of public health*, *41*(2), 125-129. https://doi.org/10.1111/1753-6405.12618 |
|  | Sandiford, P., Vivas Consuelo, D., Rouse, P., & Bramley, D. (2018). The trade-off between equity and efficiency in population health gain: Making it real. *Social science & medicine (1982)*, *212*, 136-144. https://doi.org/10.1016/j.socscimed.2018.07.005 |
|  | Santos-Neto, J. A. D., Mendes, Á. N., Pereira, A. C., & Paranhos, L. R. (2019). Assessment of health technical efficiency in the cities of the Rota dos Bandeirantes health region of the state of São Paulo, Brazil. *Ciencia & saude coletiva*, *24*(10), 3793-3803. https://doi.org/10.1590/1413-812320182410.32232017 |
|  | Sarabi Asiabar, A., Sharifi, T., Rezapour, A., Khatami Firouzabadi, S. M. A., Haghighat- Fard, P., & Mohammad-Pour, S. (2020). Technical efficiency and its affecting factors in Tehran's public hospitals: DEA approach and Tobit regression. *Medical journal of the Islamic Republic of Iran*, *34*, 176. https://doi.org/10.47176/mjiri.34.176 |
|  | Schneider, A. M., Oppel, E.-M., & Schreyögg, J. (2020). Investigating the link between medical urgency and hospital efficiency - Insights from the German hospital market. *Health care management science*, *23*(4), 649-660.  https://doi.org/10.1007/s10729-020-09520-6 |
|  | Sear, A. M. (1991). Comparison of efficiency and profitability of investor-owned multihospital systems with not-for-profit hospitals. *Health care management review*, *16*(2), 31-37. https://doi.org/10.1097/00004010-199101620-00007 |
|  | Sear, A. M. (1992). Operating characteristics and comparative performance of investor- owned multihospital systems. *Hospital & health services administration*, *37*(3), 403- 415. |
|  | Seddighi, H., Nosrati Nejad, F., & Basakha, M. (2020). Health systems efficiency in Eastern Mediterranean Region: a data envelopment analysis. *Cost effectiveness and resource allocation : C/E*, *18*, 22. https://doi.org/10.1186/s12962-020-00217-9 |
|  | See, K. F., Md Hamzah, N., & Yu, M.-M. (2021). Metafrontier efficiency analysis for hospital pharmacy services using dynamic network DEA framework [Article]. *Socio- economic planning sciences*, *78*, N.PAG-N.PAG. https://doi.org/10.1016/j.seps.2021.101044 |
|  | See, K. F., & Yen, S. H. (2018). Does happiness matter to health system efficiency? A performance analysis. *Health economics review*, *8*(1), 33. https://doi.org/10.1186/s13561-018-0214-6 |
|  | Serván-Mori, E., Chivardi, C., Mendoza, M. Á., & Nigenda, G. (2018). A longitudinal assessment of technical efficiency in the outpatient production of maternal health services in México. *Health policy and planning*, *33*(8), 888-897. https://doi.org/10.1093/heapol/czy074 |
|  | Shahhoseini, R., Tofighi, S., Jaafaripooyan, E., & Safiaryan, R. (2011). Efficiency measurement in developing countries: application of data envelopment analysis for Iranian hospitals. *Health services management research*, *24*(2), 75-80. https://doi.org/10.1258/hsmr.2010.010017 |
|  | Shaqura, I. I., Gholami, M., & Akbari Sari, A. (2021). Assessment of public hospitals performance in Gaza governorates using the Pabón Lasso Model. *The International journal of health planning and management*, *36*(4), 1223-1235. https://doi.org/10.1002/hpm.3159 |
|  | Shaqura, I. I., Gholami, M., & Sari, A. A. (2021). Evaluation of performance at Palestinian public hospitals using Pabón Lasso model. *The International journal of health planning and management*, *36*(3), 896-910. https://doi.org/10.1002/hpm.3124 |
|  | Sheikhzadeh, Y., Roudsari, A. V., Vahidi, R. G., Emrouznejad, A., & Dastgiri, S. (2012). Public and private hospital services reform using data envelopment analysis to measure technical, scale, allocative, and cost efficiencies. *Health promotion perspectives*, *2*(1), 28-41. https://doi.org/10.5681/hpp.2012.004 |
|  | Sherman, H. D. (1984). Hospital efficiency measurement and evaluation. Empirical test of a new technique. *Medical care*, *22*(10), 922-938.  https://doi.org/10.1097/00005650-198410000-00005 |
|  | Shinjo, D., Tachimori, H., Sakurai, K., Ohnuma, T., Fujimori, K., & Fushimi, K. (2017). Factors affecting prolonged length of stay in psychiatric patients in Japan: A retrospective observational study. *Psychiatry and clinical neurosciences*, *71*(8), 542- 553. https://doi.org/10.1111/pcn.12521 |
|  | Shukla, R. K., Pestian, J., & Clement, J. (1997). A comparative analysis of revenue and cost- management strategies of not-for-profit and for-profit hospitals. *Hospital & health services administration*, *42*(1), 117-134. |
|  | Siddharthan, K., Ahern, M., & Rosenman, R. (2000). Data envelopment analysis to determine efficiencies of health maintenance organizations. *Health care management science*, *3*(1), 23-29. https://doi.org/10.1023/a:1019072819828 |
|  | Sielskas, A. (2021). Determinants of hospital inefficiency. The case of Polish county hospitals. *PloS one*, *16*(8), e0256267. https://doi.org/10.1371/journal.pone.0256267 |
|  | Singh, P., Farhan, M., & Singh, P. (2020). An investigation on impact of workplace efficiency on quality of health services: A case of tertiary hospitals of Punjab [Article]. *European Journal of Molecular and Clinical Medicine*, *7*(9), 2040-2048. |
|  | Sinuany-Stern, Z., Cohen-Kadosh, S., & Friedman, L. (2016). The relationship between the efficiency of orthopedic wards and the socio-economic status of their patients [Article]. *Central European Journal of Operations Research*, *24*(4), 853-876. https://doi.org/10.1007/s10100-015-0420-9 |
|  | Sommersguter-Reichmann, M., & Stepan, A. (2015). The interplay between regulation and efficiency: Evidence from the Austrian hospital inpatient sector [Article]. *Socio- economic planning sciences*, *52*, 10-21. https://doi.org/10.1016/j.seps.2015.09.001 |
|  | Steele, R., & Gray, A. M. (1982). Statistical cost analysis: the hospital case [Article]. *Applied Economics*, *14*(5), 491. https://doi.org/10.1080/00036848200000044 |
|  | Steinmann, L., Dittrich, G., Karmann, A., & Zweifel, P. (2004). Measuring and comparing the (in)efficiency of German and Swiss hospitals. *The European journal of health economics : HEPAC : health economics in prevention and care*, *5*(3), 216-226. https://doi.org/10.1007/s10198-004-0227-4 |
|  | Sultan, W. I. M., & Crispim, J. (2018). Measuring the efficiency of Palestinian public hospitals during 2010-2015: an application of a two-stage DEA method. *BMC health services research*, *18*(1), 381. https://doi.org/10.1186/s12913-018-3228-1 |
|  | Suraratdecha, C., & Okunade, A. A. (2006). Measuring operational efficiency in a health care system: a case study from Thailand. *Health policy (Amsterdam, Netherlands)*, *77*(1), 2-23. https://doi.org/10.1016/j.healthpol.2005.07.005 |
|  | Takundwa, R., Jowett, S., McLeod, H., & Peñaloza-Ramos, M. (2017). The Effects of Environmental Factors on the Efficiency of Clinical Commissioning Groups in England: A Data Envelopment Analysis. *Journal of medical systems*, *41*(6), 1-7. https://doi.org/10.1007/s10916-017-0740-5 |
|  | Tanaka, M., Lee, J., Ikai, H., & Imanaka, Y. (2013). Development of efficiency indicators of operating room management for multi-institutional comparisons. *Journal of evaluation in clinical practice*, *19*(2), 335-341.  https://doi.org/10.1111/j.1365-2753.2012.01829.x |
|  | Thai, Q. K., & Noguchi, M. (2020). Measuring Efficiency of the New Zealand District Health Boards: An Empirical Research Using Two‐Stage Data Envelopment Analysis [Article]. *Economic Papers*, *39*(1), 58-71. https://doi.org/10.1111/1759-3441.12269 |
|  | Tiemann, O., & Schreyögg, J. (2009). Effects of Ownership on Hospital Efficiency in Germany [Article]. *Business Research*, *2*(2), 115-145. https://doi.org/10.1007/BF03342707 |
|  | Tigga, N. S., & Rajan, S. I. (2016). Efficiency of Health System in Four States of India : A District Level Analysis [Article]. *Productivity*, *56*(4), 9-23. |
|  | Tlotlego, N., Nonvignon, J., Sambo, L. G., Asbu, E. Z., & Kirigia, J. M. (2010). Assessment of productivity of hospitals in Botswana: a DEA application. *International archives of medicine*, *3*, 27. https://doi.org/10.1186/1755-7682-3-27 |
|  | Usherwood, T. P. (1987). Clinical efficiency in general practice. *Family practice*, *4*(2), 149- 151. https://doi.org/10.1093/fampra/4.2.149 |
|  | Valdmanis, V. G., Rosko, M. D., Leleu, H., & Mukamel, D. B. (2017). Assessing overall, technical, and scale efficiency among home health care agencies. *Health care management science*, *20*(2), 265-275. https://doi.org/10.1007/s10729-015-9351-1 |
|  | van Ineveld, M., van Oostrum, J., Vermeulen, R., Steenhoek, A., & van de Klundert, J. (2016). Productivity and quality of Dutch hospitals during system reform. *Health care management science*, *19*(3), 279-290. https://doi.org/10.1007/s10729-015-9321-7 |
|  | Varabyova, Y., Blankart, C. R., Torbica, A., & Schreyögg, J. (2017). Comparing the Efficiency of Hospitals in Italy and Germany: Nonparametric Conditional Approach Based on Partial Frontier. *Health care management science*, *20*(3), 379-394. https://doi.org/10.1007/s10729-016-9359-1 |
|  | Varela, P. S., & de Andrade Martins, G. (2011). Efficiency of Primary Health Care Spending by Municipalities in the Metropolitan Region of São Paulo: A Comparative Analysis of DEA Models [Article]. *Review of Business*, *32*(1), 17-34. |
|  | Vaz, F. S., Ferreira, A. M., Kulkarni, M. S., & Motghare, D. D. (2007). Bed utilization indices at a tertiary care hospital in Goa: an eight year trend analysis. *Indian journal of public health*, *51*(4), 231-233. |
|  | Vera, A., & Kuntz, L. (2007). Process-based organization design and hospital efficiency. *Health care management review*, *32*(1), 55-65. https://doi.org/10.1097/00004010-200701000-00008 |
|  | W. D. S, A., Juni, M. H., Lim Poh, Y., Ghani, J. A., & Kamarudin, F. (2019). TECHNICAL EFFICIENCY ASSESSMENT OF MATERNAL HEALTH SERVICES IN NEGERI SEMBILAN, MALAYSIA USING DATA ENVELOPMENT ANALYSIS. *International Journal of Public Health & Clinical Sciences (IJPHCS)*, *6*(4), 79-95. https://doi.org/10.32827/ijphcs.6.4.79 |
|  | Walker, D. M. (2018). Does participation in health information exchange improve hospital efficiency? *Health care management science*, *21*(3), 426-438. https://doi.org/10.1007/s10729-017-9396-4 |
|  | Wan, T. T. H., Lin, B. Y.-J., & Ma, A. (2002). Integration mechanisms and hospital efficiency in integrated health care delivery systems. *Journal of medical systems*, *26*(2), 127-143. https://doi.org/10.1023/a:1014805909707 |
|  | Wang, L., Grignon, M., Perry, S., Chen, X.-K., Ytsma, A., Allin, S., & Gapanenko, K. (2018). The Determinants of the Technical Efficiency of Acute Inpatient Care in Canada. *Health services research*, *53*(6), 4829-4847. https://doi.org/10.1111/1475-6773.12861 |
|  | Wang, M.-L., Fang, H.-Q., Tao, H.-B., Cheng, Z.-H., Lin, X.-J., Cai, M., Xu, C., & Jiang, S. (2017). Bootstrapping data envelopment analysis of efficiency and productivity of county public hospitals in Eastern, Central, and Western China after the public hospital reform. *Journal of Huazhong University of Science and Technology. Medical sciences = Hua zhong ke ji da xue xue bao. Yi xue Ying De wen ban = Huazhong keji daxue xuebao. Yixue Yingdewen ban*, *37*(5), 681-692.  https://doi.org/10.1007/s11596-017-1789-6 |
|  | Wang, X., Luo, H., Qin, X., Feng, J., Gao, H., & Feng, Q. (2016). Evaluation of performance and impacts of maternal and child health hospital services using Data Envelopment Analysis in Guangxi Zhuang Autonomous Region, China: a comparison study among poverty and non-poverty county level hospitals. *International journal for equity in health*, *15*(1), 131. https://doi.org/10.1186/s12939-016-0420-y |
|  | Watcharasriroj, B., & Tang, J. C. S. (2004). The effects of size and information technology on hospital efficiency [Article]. *Journal of High Technology Management Research*, *15*(1), 1. https://doi.org/10.1016/j.hitech.2003.09.001 |
|  | Werblow, A. (2005). [Efficiency of general practice in Switzerland]. *Gesundheitswesen (Bundesverband der Arzte des Offentlichen Gesundheitsdienstes (Germany))*, *67*(2), 107-111. https://doi.org/10.1055/s-2005-857895 |
|  | White, K. R., & Ozcan, Y. A. (1996). Church ownership and hospital efficiency. *Hospital & health services administration*, *41*(3), 297-310. |
|  | Wilson, A. B., Kerr, B. J., Bastian, N. D., & Fulton, L. V. (2012). Financial performance monitoring of the technical efficiency of critical access hospitals: a data envelopment analysis and logistic regression modeling approach. *Journal of healthcare management / American College of Healthcare Executives*, *57*(3), 200- 212. |
|  | Wranik, D. (2012). Healthcare policy tools as determinants of health-system efficiency: evidence from the OECD. *Health economics, policy, and law*, *7*(2), 197-226. https://doi.org/10.1017/S1744133111000211 |
|  | Wu, C.-H., Chang, C.-C., Chen, P.-C., & Kuo, K.-N. (2013). Efficiency and productivity change in Taiwan's hospitals: a non-radial quality-adjusted measurement [Article]. *Central European Journal of Operations Research*, *21*(2), 431-453. https://doi.org/10.1007/s10100-012-0238-7 |
|  | Wu, C.-H., Chang, C.-C., & Kuo, K. N. (2008). Evaluating the Resource Allocation Efficiency of the Healthcare System in Taiwan. *International Journal of Public Policy*, *3*(5-6), 403-418. https://doi.org/http://www.inderscience.com/ijpp |
|  | Xu, G.-C., Zheng, J., Zhou, Z.-J., Zhou, C.-K., & Zhao, Y. (2015). Comparative Study of Three Commonly Used Methods for Hospital Efficiency Analysis in Beijing Tertiary Public Hospitals, China. *Chinese medical journal*, *128*(23), 3185-3190. https://doi.org/10.4103/0366-6999.170279 |
|  | Yankovsky, A., Gajewski, B. J., & Dunton, N. (2016). Trends in Nursing Care Efficiency From 2007 to 2011 on Acute Nursing Units. *Nursing economic$*, *34*(6), 266-276. |
|  | Yi, M., Peng, J., Zhang, L., & Zhang, Y. (2020). Is the allocation of medical and health resources effective? Characteristic facts from regional heterogeneity in China. *International journal for equity in health*, *19*(1), 89.  https://doi.org/10.1186/s12939-020-01201-8 |
|  | Yildiz, M. S., Heboyan, V., & Khan, M. M. (2018). Estimating technical efficiency of Turkish hospitals: implications for hospital reform initiatives. *BMC health services research*, *18*(1), 401. https://doi.org/10.1186/s12913-018-3239-y |
|  | Yin, G., Chen, C., Zhuo, L., He, Q., & Tao, H. (2021). Efficiency Comparison of Public Hospitals under Different Administrative Affiliations in China: A Pilot City Case. *Healthcare (Basel, Switzerland)*, *9*(4). https://doi.org/10.3390/healthcare9040437 |
|  | Yitbarek, K., Abraham, G., Adamu, A., Tsega, G., Berhane, M., Hurlburt, S., Mann, C., & Woldie, M. (2019). Technical efficiency of neonatal health services in primary health care facilities of Southwest Ethiopia: a two-stage data envelopment analysis. *Health economics review*, *9*(1), 27. https://doi.org/10.1186/s13561-019-0245-7 |
|  | Yitbarek, K., Abraham, G., Berhane, M., Hurlburt, S., Mann, C., Adamu, A., Tsega, G., & Woldie, M. (2021). Significant inefficiency in running community health systems: The case of health posts in Southwest Ethiopia. *PloS one*, *16*(2), e0246559. https://doi.org/10.1371/journal.pone.0246559 |
|  | Yitbarek, K., Adamu, A., Tsega, G., Siraneh, Y., Erchafo, B., Yewhalaw, D., Tekle, F., & Woldie, M. (2019). Technical Efficiency of Maternal and Reproductive Health Services in Public Hospitals of Oromia Regional State, Ethiopia. *Health services insights*, *12*, 1178632919837630. https://doi.org/10.1177/1178632919837630 |
|  | Yuan, H., Li, H., & Hou, Z. (2020). Is it worth outsourcing essential public health services in China?-Evidence from Beilin District of Xi'an. *The International journal of health planning and management*, *35*(6), 1486-1502. https://doi.org/10.1002/hpm.3051 |
|  | Yue, J., Zhou, Y., Ning, J., Yin, G., Wu, Y., Li, J., & Tao, H. (2021). Efficiency and productivity of county-level traditional Chinese medicine hospitals in Hubei Province, China: A retrospective study based on 17 years of panel data. *The International journal of health planning and management*, *36*(4), 1308-1325. https://doi.org/10.1002/hpm.3171 |
|  | Yusefzadeh, H., Ghaderi, H., Bagherzade, R., & Barouni, M. (2013). The efficiency and budgeting of public hospitals: case study of iran. *Iranian Red Crescent medical journal*, *15*(5), 393-399. https://doi.org/10.5812/ircmj.4742 |
|  | Zaim, S., Bayyurt, N., Turkyilmaz, A., Solakoglu, N., & Zaim, H. (2007). Measuring and Evaluating Efficiency of Hospitals Through Total Quality Management: A Multi- Criteria Data Envelopment Analysis Model [Article]. *Journal of Transnational Management*, *12*(4), 77-97. https://doi.org/10.1300/J482v12n04_05 |
|  | Zamo-Akono, C., Ndjokou, M. M., & Song-Ntamack, S. (2013). Institutions and Hospital Efficiency in Cameroon: A Data Envelopment Analysis. *Journal of African Development*, *15*(1), 45-71.  https://doi.org/http://www.jadafea.com/previous-issues/ |
|  | Zarulli, V., Sopina, E., Toffolutti, V., & Lenart, A. (2021). Health care system efficiency and life expectancy: A 140-country study. *PloS one*, *16*(7), e0253450. https://doi.org/10.1371/journal.pone.0253450 |
|  | Zavras, A. I., Tsakos, G., Economou, C., & Kyriopoulos, J. (2002). Using DEA to evaluate efficiency and formulate policy within a Greek national primary health care network. Data Envelopment Analysis. *Journal of medical systems*, *26*(4), 285-292. https://doi.org/10.1023/a:1015860318972 |
|  | Zere, E., Mbeeli, T., Shangula, K., Mandlhate, C., Mutirua, K., Tjivambi, B., & Kapenambili, W. (2006). Technical efficiency of district hospitals: evidence from Namibia using data envelopment analysis. *Cost effectiveness and resource allocation: C/E*, *4*, 5. https://doi.org/10.1186/1478-7547-4-5 |
|  | Zere, E., McIntyre, D., & Addison, T. (2001). TECHNICAL EFFICIENCY AND PRODUCTIVITY OF PUBLIC SECTOR HOSPITALS IN THREE SOUTH AFRICAN PROVINCES [Article]. *South African Journal of Economics*, *69*(2), 336. |
|  | Zhang, L., Zeng, Y., & Fang, Y. (2019). Evaluating the technical efficiency of care among long-term care facilities in Xiamen, China: based on data envelopment analysis and Tobit model. *BMC public health*, *19*(1), N.PAG-N.PAG.  https://doi.org/10.1186/s12889-019-7571-x |
|  | Zhang, N., Hu, A., & Zheng, J. (2007). Using Data Envelopment Analysis Approach to Estimate the Health Production Efficiencies in China. *Frontiers of Economics in China*, *2*(1), 1- 23. |
|  | Zhang, T., Lu, W., & Tao, H. (2020). Efficiency of health resource utilisation in primary- level maternal and child health hospitals in Shanxi Province, China: a bootstrapping data envelopment analysis and truncated regression approach. *BMC health services research*, *20*(1), 179. https://doi.org/10.1186/s12913-020-5032-y |
|  | Zheng, D., Gong, J., & Zhang, C. (2019). Efficiency of medical service systems in the rural areas of Mainland China: a comparative study from 2013 to 2017. *Public health*, *171*, 139-147. https://doi.org/10.1016/j.puhe.2019.04.003 |
|  | Zheng, W., Sun, H., Zhang, P., Zhou, G., Jin, Q., & Lu, X. (2018). A four-stage DEA-based efficiency evaluation of public hospitals in China after the implementation of new medical reforms. *PloS one*, *13*(10), e0203780. https://doi.org/10.1371/journal.pone.0203780 |
|  | Zuckerman, S., Hadley, J., & Iezzoni, L. (1994). Measuring hospital efficiency with frontier cost functions. *Journal of health economics*, *13*(3), 255-280. https://doi.org/10.1016/0167-6296(94)90027-2 |
